# Free Energy, Value, and Attractors

**DOI:** 10.1155/2012/937860

**Published:** 2011-12-21

**Authors:** Karl Friston, Ping Ao

**Affiliations:** ^1^The Wellcome Trust Centre for Neuroimaging, UCL, Institute of Neurology, 12 Queen Square, London WC1N 3BG, UK; ^2^Shanghai Center for Systems Biomedicine, Key Laboratory of Systems Biomedicine of Ministry of Education, Shanghai Jiao Tong University, Shanghai 200240, China; ^3^Departments of Mechanical Engineering and Physics, University of Washington, Seattle, WA 98195, USA

## Abstract

It has been suggested recently that action and perception can be understood as minimising the free energy of sensory samples. This ensures that agents sample the environment to maximise the evidence for their model of the world, such that exchanges with the environment are predictable and adaptive. However, the free energy account does not invoke reward or cost-functions from reinforcement-learning and optimal control theory. We therefore ask whether reward is necessary to explain adaptive behaviour. The free energy formulation uses ideas from statistical physics to explain action in terms of minimising sensory surprise. Conversely, reinforcement-learning has its roots in behaviourism and engineering and assumes that agents optimise a policy to maximise future reward. This paper tries to connect the two formulations and concludes that optimal policies correspond to empirical priors on the trajectories of hidden environmental states, which compel agents to seek out the (valuable) states they expect to encounter.

## 1. Introduction

This paper is about the emergence of adaptive behaviour in agents or phenotypes immersed in an inconstant environment. We will compare and contrast two perspectives; one based upon a free energy principle [1] and the other on optimal control and reinforcement-learning [2–5]. The key difference between these perspectives rests on what an agent optimises. The free energy principle assumes that both the action and internal states of an agent minimise the surprise (the negative log-likelihood) of sensory states. This surprise does not have to be learned because it defines the agent. In brief, being a particular agent induces a probability density on the states it can occupy (e.g., a fish in water) and, implicitly, surprising states (e.g., a fish out of water). Conversely, in reinforcement-learning, agents try to optimise a policy that maximises expected reward. We ask how free energy and policies are related and how they specify adaptive behaviour. Our main conclusion is that policies can be cast as beliefs about the state-transitions that determine free energy. This has some important implications for understanding the quantities that the brain has to represent when responding adaptively to changes in the sensorium.

We have shown recently that adaptive behaviour can be prescribed by prior expectations about sensory inputs, which action tries to fulfill [6]. This is called *active inference* and can be implemented, in the context of supervised learning, by exposing agents to an environment that enforces desired motion through state-space [7]. These trajectories are learned and recapitulated in the absence of supervision. The resulting behaviour is robust to unexpected or random perturbations and can be used to solve benchmark problems in reinforcement-learning and optimal control: see [7] for a treatment of the mountain-car problem. Essentially, active inference replaces value-learning with perceptual learning that optimises empirical (acquired) priors in the agent's internal model of its world. These priors specify the free energy associated with sensory signals and guide action to ensure sensations conform to prior beliefs. In this paper, we consider the harder problem addressed by reinforcement-learning and other semisupervised schemes. These schemes try to account for adaptive behaviour, given only a function that labels states as attractive or costly. This means agents have to access distal attractors, under proximal constraints furnished by the environment and their repertoire of allowable actions. We will take a dynamical perspective on this problem, which highlights the relationship between active inference and reinforcement-learning and the connection between empirical priors and policies.

This paper comprises five sections. The first considers adaptive behaviour in terms of equilibria and random attractors, which we attempt to link later to concepts in behavioural economics and optimal decision or game theory [8, 9]. This section considers autopoietic (self-creating) attractors to result from minimising the conditional entropy (average surprise) of an agent's states through action. However, agents can only infer hidden states of the environment given their sensory states, which means agents must minimise the surprise associated with sensations. The second section shows how agents can do this using an upper (free energy) bound on sensory surprise. This leads to a free energy formulation of well-known inference and learning schemes based on generative models of the world [10–13]. In brief, the imperatives established in the first section are satisfied when action and inference minimise free energy. However, the principle of minimising free energy also applies to the form of the generative model entailed by an agent (its formal priors). These encode prior beliefs about the transitions or motion of hidden states and ensuing attractors, which action tries to fulfil. These priors or policies are considered from a dynamical perspective in the remaining sections. Section three considers some universal policies, starting with the Helmholtz decomposition and introducing the notion of value, detailed balance, and divergence-free flow. The final two sections look at *fixed-point* and *itinerant* polices, respectively. Fixed-point policies attract trajectories to (low-cost) points in state-space. These policies are considered in reinforcement-learning and optimal control theory [2, 4, 14]. They are based on Lyapunov (value) functions that specify the policy. However, under the Helmholtz decomposition, value functions are an incomplete specification of policies. This speaks to more general forms of (itinerant) policies that rest on the autovitiation (self-destruction) of costly attractors and itinerant (wandering or searching) motion through state-space. We illustrate the basic ideas using the same mountain-car problem that we have used previously in the context of supervised learning [7].

The main conclusion of this paper is that it is sufficient to minimise the average surprise (conditional entropy) of an agent's states to explain adaptive behaviour. This can be achieved by policies or empirical priors (equations of motion) that guide action and induce random attractors in its state-space. These attract agents to (low-cost) invariant sets of states and lead to autopoietic and ergodic behaviour. 

## 2. Ensemble Dynamics and Random Attractors

What do adaptive agents optimise? We address this question using an ensemble density formulation, which has close connections to models of evolutionary processes [15–17] and equilibria in game theory [18]. We also introduce a complementary perspective based on random dynamical systems [19]. The equilibrium approach rests on an ensemble density over the states of an agent. This can be regarded as the density of innumerable copies of the agent, each represented by a point in phase or state-space. This density is essentially a probabilistic definition of the agent, in terms of the states it occupies. For a well-defined agent to exist its ensemble density must be *ergodic*; that is, an invariant probability measure [20]. In other words, the density cannot change over time; otherwise, the definition of an agent (in terms of the states it occupies) would change. A simple example here would be the temperature of an organism, whose ensemble density is confined to certain phase-boundaries. Transgressing these boundaries would change the agent into something else (usually a dead agent). The simple fact that an agent's ensemble density exists and is confined within phase-boundaries (i.e., is ergodic or invariant) has some fundamental implications, which we now consider more formally.

### 2.1. Set Up: States and Dependencies

If an agent and its environment have states, what does it mean for the states of an agent to be distinct from those of its environment? We will take this to mean that an agent has *internal* and *external* states that are conditionally independent and are therefore separated by a Markov blanket. The minimal (nontrivial) requirement for this blanket to exist is a partition of the states into two pairs of subsets, where one pair constitutes a Markov blanket for the other. 

**Figure 1:**
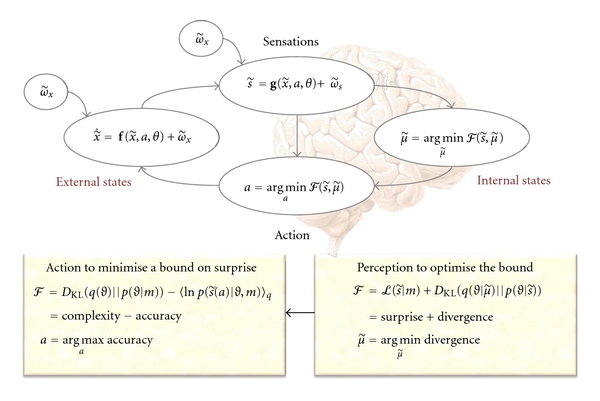
The free energy principle. The schematic shows the probabilistic dependencies (arrows) among the quantities that define free energy. These include the internal states of the brain $\tilde{\mu }(t)$ and quantities describing its exchange with the environment. These are the generalized sensory states $\tilde{s}(t)={\left[s,s^{\prime },s^{\prime \prime },\ldots \right]}^{T}$ and action *a*(*t*). The environment is described by equations of motion, which specify the trajectory of its hidden states and a mapping to sensory states. The quantities $\vartheta ⊃(\tilde{x},\theta )$ causing sensory states comprise hidden states and parameters. The hidden parameters control the equations (**f**, **g**) and precision (inverse variance) of random fluctuations (*ω*_*x*_(*t*), *ω*_*s*_(*t*)) on hidden and sensory states. Internal brain states and action minimize free energy $ℱ(\tilde{s},\tilde{\mu })$, which is a function of sensory states and a probabilistic representation $q(\vartheta |\tilde{\mu })$ of their causes. This representation is called the recognition density and is encoded by internal states that play the role of sufficient statistics. The free energy depends on two probability densities; the recognition density, $q(\vartheta |\tilde{\mu })$, and one that generates sensory samples and their causes, $p(\tilde{s},\vartheta |m)$. The latter represents a probabilistic generative model (denoted by *m*), whose form is entailed by the agent. The lower panels provide alternative expressions for the free energy to show what its minimization entails. Action can only reduce free energy by increasing accuracy (i.e., selectively sampling sensory states that are predicted). Conversely, optimizing internal states makes the representation an approximate conditional density on the causes of sensory states. This enables action to avoid surprising sensory encounters. See main text for further details.

This straightforward consideration suggests a four-way partition of state-space *X* × *S* × *A* × *M* ⊂ ℝ associated with an agent *m* ∈ *ℳ*. Here, external states $\tilde{x}\in X$ represent states of the agent's immediate environment, such as forces, temperature, and physiological states. The tilde notion denotes a generalised state, which includes temporal derivatives to arbitrarily high order, such that $\tilde{x}={\left[x,x^{\prime },x^{\prime \prime },\ldots \right]}^{T}$ comprises position, velocity, acceleration, jerk, and so on. The internal states $\tilde{\mu }\in M$ correspond to things like intracellular concentrations, neuronal activity, and so forth. We will see later that these are internal representations of external states. These states are separated from each other by a Markov blanket *S* × *A*, comprising *sensory* states that mediate the influence of external states on internal states and *action*, which mediates the influence of internal states on external states. Sensory states $\tilde{s}\in S$, like photoreceptor activity, depend on external states, while action *a* ∈ *A*, like alpha motor neuron activity, depends on internal states.  Figure 1 illustrates these conditional dependencies in terms of a graphical model, in which action and sensation form a Markov blanket separating external and internal states. In other words, external states are "hidden" from the agent's internal states. We will therefore refer to external states as *hidden* states. 

The notion of a Markov blanket refers to a (statistical) boundary between the internal and hidden states of an agent. For simple (cellular) organisms, this could be associated with the cell surface, where sensory states correspond to the states of receptors and ion channels and action to various transporter and cell adhesion processes. For more complicated multicellular organisms (like us) the boundary of an agent is probably best thought of in terms of systems. For example, neuronal systems have clearly defined sensory states at their receptors and action is mediated by a discrete number of effectors. Here, the notion of a surface is probably less useful, in the sense that the spatial deployment of sensory epithelia becomes a hidden state (and depends on action).

The external state-space we have in mind is high dimensional, covering the myriad of macroscopic states that constitute an embodied agent and its proximal environment. We assume that this system is open and that its states are confined to a low-dimensional manifold *𝒪* ⊂ *X* that endow the agent with attributes. More precisely, the agent has *observables* (i.e., phenotypic traits or characteristics) that are given by real-valued functions, whose domain is the bounded set *𝒪* ⊂ *X*. This implies that there are states $\tilde{x}\notin 𝒪$ an agent cannot occupy (e.g., very low temperatures). An observable is a property of the state that can be determined by some operator. A simple example of a bounded operator would be length, which must be greater than zero.

The existence of macroscopic states appeals to the fact that interactions among microscopic states generally lead to macroscopic order. There are many examples of this in the literature on complex systems and self-organisation. Key examples of macroscopic states are the order parameters used to describe phase-transitions [21]. The order parameter concept has been generalized to the slaving principle [22], under which the fast (stable) dynamics of rapidly dissipating patterns (modes or phase-functions) of microscopic states are determined by the slow (unstable) dynamics of a few macroscopic states (order parameters). These states can be regarded as the amplitudes of patterns that determine macroscopic behaviour. The enslaving of stable patterns by macroscopic states greatly reduces the degrees of freedom of the system and leads to the emergence of macroscopic order (e.g., pattern formation). A similar separation of temporal scales is seen in centre manifold theory [23]. See [24–26] for interesting examples and applications. We will assume that macroscopic states $\tilde{x}\in X$ are (unique phase) functions of the microscopic states that they enslave.

**Figure 2:**
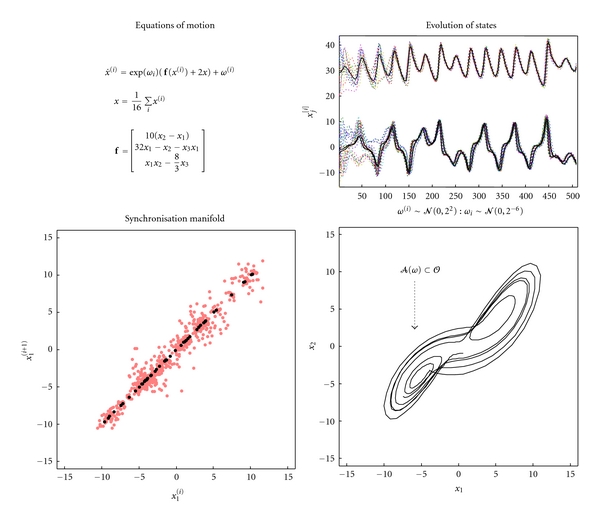
Self-organisation and the emergence of macroscopic behaviour. This figure shows a simple example of self-organisation using sixteen (Lorenz) oscillators that have been coupled to each other, so that each oscillator (with three microscopic states) sees the other oscillators. This is an example of a globally coupled map, where the dynamics of each oscillator conform to a classical Lorenz system. The equations of motion are provided in the left panel for each microstate, *x*_*j*_^(*i*)^ : *i* ∈ 1,…, 16 : *j* ∈ 1,  2,  3, whose average constitutes a macrostate *x*_*j*_ : *j* ∈ 1,  2,  3. Each oscillator has its own random fluctuations *ω* ^(*i*)^(*t*) ∈ ℝ and speed exp⁡(*ω*_*i*_) ∈ ℝ^+^. The upper right panel shows the evolution of the microstates (dotted lines) and the macrostates (solid lines) over 512 time steps of one 1/32 second. The lower right panel, shows the first two macrostates plotted against each other to show the implicit attractor that emerges from self-organisation. The lower left panel shows the implicit synchronisation manifold by plotting the first states from successive pairs of oscillators (pink) and their averages (black) against each other. This simulation used low levels of noise on the motion of the microstates *ω* ^(*i*)^ ~ *𝒩*(0, 2^2^) and the log-rate constants *ω*_*i*_ ~ *𝒩*(0, 2^−6^) that disperse the speeds of each oscillator. The initial states were randomised by sampling from a Gaussian distribution with a standard deviation of eight.

**Figure 3:**
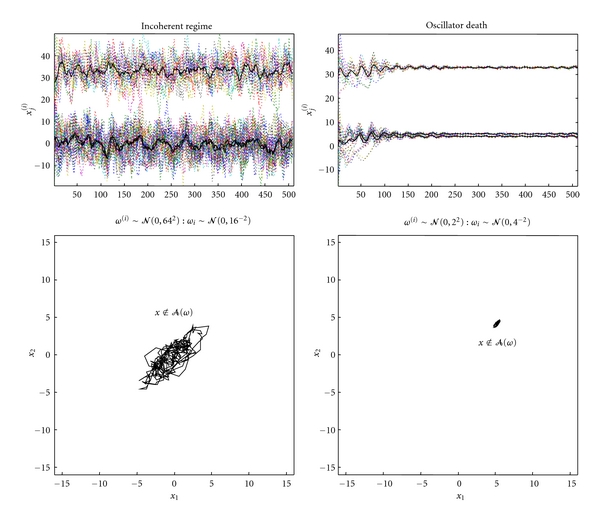
The loss of macroscopic order and oscillator death. This figure uses the same format and setup as in the previous figure but here shows the loss of macroscopic order through incoherence (left) and oscillator death (right). Incoherence was induced by increasing the random fluctuations on the motion of states to *ω* ^(*i*)^ ~ *𝒩*(0, 2^10^). Oscillator death was induced by increasing the random dispersion of speeds along each oscillators orbit to *ω*_*i*_ ~ *𝒩*(0, 2^−4^), see [24]. The ensuing macroscopic states (lower panels) now no longer belong to the attracting set of the previous figure: *𝒜*(*ω*) ⊂ *𝒪*.

The emergence of macroscopic order (and its associated states) is easy to simulate.  Figure 2 provides a simple example where sixteen (Lorenz) oscillators have been coupled to each other, so that each oscillator (with three microscopic states) sees all the other oscillators. In this example, the macroscopic states (c.f. order parameters) are just the average of each state over oscillators; this particular phase-function is known as a mean field: see [27] for a discussion of mean field treatments of neuronal dynamics. Here, the mean field enslaves the states of each oscillator so that the difference between each microscopic state and its average decays quickly; these differences are the stable patterns and decay to zero. This draws the microscopic states to a low- (three-) dimensional manifold, known as a synchronisation manifold [28]. Although the emergence of order is easy to simulate, it is also easy to destroy.  Figure 3 shows how macroscopic order collapses when the random fluctuations on the motion of states are increased. Here, there is no slaving because the system has moved from a coherent regime to an incoherent regime, where each oscillator pursues its own path. Order can also be destroyed by making the coherence trivial; this is known as oscillator death and occurs when each oscillator approaches a fixed-point in state-space (interestingly these fixed-points are unstable when the oscillators are uncoupled, see [24]). Oscillator death is illustrated in Figure 3 by increasing the random dispersion of speeds along each oscillators orbit (trajectory). In these examples, macroscopic order collapses into incoherent or trivially coherent dynamics. We have deliberately chosen to illustrate these phenomena with a collection of similar oscillators (known technically as a globally coupled map; see also [29]), because the macroscopic dynamics recapitulate the dynamics of each oscillator in isolation. This means one could imagine that the microscopic states are themselves phase-functions of micromicroscopic states and so on *ad infinitum*. Heuristically, this speaks to the hierarchical and self-similar dynamics of complex self-organising systems [30, 31].

In summary, the emergence of macroscopic order is not mysterious and arises from a natural separation of temporal scales that is disclosed by some transformation of variables. However, the ensuing order is delicate and easily destroyed. In what follows, we shall try to understand how self-organisation keeps the macroscopic states of an agent within a bounded set *𝒪* ⊂ *X* for extended periods of time. To do this we will look more closely at their dynamics.

### 2.2. Dynamics and Ergodicity

Let the conditional dependencies among the (macroscopic) states *X* × *S* × *A* × *M* ⊂ ℝ in Figure 1 be described by the following coupled differential equations:

(1) $$\begin{array}{c} \dot{\tilde{x}}=f\left(\tilde{x},a,\theta \right)+{\tilde{\omega }}_{a},\\ \tilde{s}=g\left(\tilde{x},a,\theta \right)+{\tilde{\omega }}_{s}, \end{array}$$

where (as we will see later)

(2) $$\begin{array}{c} \dot{a}=-\partial_{a}ℱ\left(\tilde{s},\tilde{\mu }\right),\\ \dot{\tilde{\mu }}=-\partial_{\tilde{\mu }}ℱ\left(\tilde{s},\tilde{\mu }\right)+𝒟\tilde{\mu }. \end{array}$$

Here, *𝒟* is a derivative matrix operator with identity matrices along its first diagonal such that $𝒟\tilde{\mu }={\left[\mu^{\prime },\mu^{\prime \prime },\mu^{\prime \prime \prime },\ldots \right]}^{T}$. The first (stochastic differential) equation above describes the flow of hidden states in terms of a mapping **f** : *X* × *A* → *X* and some random fluctuations, ${\tilde{\omega }}_{a}\in \Omega_{a}$, while the second expresses sensory states in terms of a sensory mapping **g** : *X* → *S* and noise, ${\tilde{\omega }}_{s}\in \Omega_{s}$. In this formulation, sensations are a noisy map of hidden states that evolve as a function of themselves and action, where exogenous influences from outside the proximal environment are absorbed into the random fluctuations. The quantities *θ* represent time-invariant parameters of the equations of motion and sensory mapping. For simplicity, we will omit *θ* for the remainder of this section and return to them later. The second pair of equations describes action *a* : *M* × *S* → *A* and internal states $\tilde{\mu }:M\times S\to M$ as a gradient descent on a functional (function of a function) of sensory and internal states: $ℱ(\tilde{s},\tilde{\mu })\in \mathbb{R}$. The purpose of this paper is to motivate the nature of this (free energy) functional and relate it to classical treatments of optimal behaviour. 

As it stands, (1) is difficult to analyse because flow is a nonautonomous function of action. We can finesse this (without loss of generality) by expressing action as a function of the current state $u(\tilde{x}(t))$ plus a fluctuating part *ω*_*u*_(*t*) using a Taylor expansion around the action expected in state $\tilde{x}\in X$

(3) $$\begin{array}{cc} \dot{\tilde{x}} & =f\left(\tilde{x},a\right)+{\tilde{\omega }}_{a}\\ & =f\left(\tilde{x},u\right)+{\tilde{\omega }}_{x}\\ {\tilde{\omega }}_{x} & ={\tilde{\omega }}_{a}+\partial_{u}f\cdot \omega_{u}+\cdots \\ a\left(t\right) & =u\left(\tilde{x}\right)+\omega_{u}. \end{array}$$

Equation (3) reformulates the dynamics in terms of controlled flow $f(\tilde{x},u):=f:X\to X$ and controlled fluctuations ${\tilde{\omega }}_{x}\in \Omega_{x}$. This formulation is autonomous in the sense that controlled flow depends only on the current state. Furthermore, it allows us to connect to the optimal control literature that usually assumes *control* $u(\tilde{x})$ is a function of, and only of, the current state. In our setup, control is the expected (average) action in a hidden state. In contrast, action *a* : *M* × *S* → *A* depends on internal and sensory states and therefore depends upon hidden states and random fluctuations in the past. In what follows, we will refer to controlled flow as a *policy* in the sense that it describes motion through state-space or transitions among states, in the absence of random effects. The policy is also the expected flow because it is the flow under expected action.

With these variables in place we can now ask what can be deduced about the nature of action and control, given the existence of agents. Our starting point is that agents are ergodic [20, 32], in the sense that their ensemble density is invariant (conserved) over a suitably long time scale. This is just another way of saying that agents occupy a subset of states *𝒪* ⊂ *X* for long periods of time. The implicit ergodic (invariant) density $p(\tilde{x}|m):=p(\tilde{x},\infty |m)$ is the stationary solution to the Fokker-Planck equation (also known as the Kolmogorov forward equation; [33]) describing the dynamics of the ensemble density over hidden states

(4) $$\begin{array}{c} \dot{p}\left(\tilde{x},t\;|\;m\right)=\Lambda p:=\nabla \cdot \Gamma \nabla p-\nabla \cdot \left(fp\right)\\ \dot{p}\left(\tilde{x}\;|\;m\right)=0\Rightarrow \\ p\left(\tilde{x}\;|\;m\right)=ℰ\left(\Lambda \right). \end{array}$$

Here, Λ(**f**, Γ) is the Fokker-Planck operator and Γ is half the covariance (amplitude) of the controlled fluctuations (a.k.a. the diffusion tensor). Equation (4) assumes the fluctuations are temporally uncorrelated (Wiener) processes; however, because the fluctuations ${\tilde{\omega }}_{x}(t)$ are in generalised coordinates of motion, the fluctuations on states *per se* can be smooth and analytic [34]. The Fokker-Planck equation exploits the fact that the ensemble (probability mass) is conserved. The first (diffusion) term of the Fokker-Planck operator reflects dispersion due to the fluctuations that smooth the density. The second term describes the effects of flow that translates probability mass. The ergodic density $p:=p(\tilde{x}|m)=ℰ(\Lambda )$ is the principal eigensolution of the Fokker-Planck operator (with an eigenvalue of zero: Λ*ℰ* = 0). Crucially, this density depends only on flow and the amplitude of the controlled fluctuations. 

The ergodic density at any point in state-space is also the *sojourn* time that an individual spends there. Similarly, its conditional entropy or ensemble average of *surprise* (also known as self-information or surprisal) is the long-term average of surprise an individual experiences. The entropy and surprise associated with the hidden states are (in the long term: *T* → *∞*):

(5) $$\begin{array}{cc} ℋ\left(X\;|\;m\right) & =-\int_{X}p\left(\tilde{x}\;|\;m\right)\ln p\left(\tilde{x}\;|\;m\right)dx=\frac{1}{T}\overset{T}{\underset{0}{\int }}dtℒ\left(\tilde{x}\left(t\right)\right)\\ ℒ\left(\tilde{x}\left(t\right)\right) & =-\ln p\left(\tilde{x}\left(t\right)\;|\;m\right). \end{array}$$

The conditional entropy is an *equivocation* because it is conditioned on the agent. It is important not to confuse the conditional entropy *ℋ*(*X* | *m*) with the entropy *ℋ*(*X*): A system with low entropy may have a very high conditional entropy unless it occupies states that are characteristic of the agent (because $p(\tilde{x}\left(t\right)|m)$ will be persistently small). We will use these characterisations of the ergodic density extensively below and assume that they are all conditional. Readers with a physics background will note that surprise can be regarded as a Lagrangian, with a path-integral $\int dtℒ(\tilde{x}(t))=Tℋ(X|m)$ that is proportional to entropy. We will call on this equivalence later. In this paper, Lagrangians are negative log-probabilities or surprise.

The terms entropy and surprise are used here in an information theoretic (Shannon) sense. From a thermodynamic perceptive, the ergodic density corresponds to a *steady state*, in which (biological) agents are generally far from thermodynamic equilibrium; even though the ensemble density on their macroscopic states (e.g., intracellular concentrations) is stationary. In computational biology, the notion of *nonequilibrium steady state* is central to the study of the homeostatic cellular biochemistry of microscopic states. In this context, the chemical master equation plays the same role as the Fokker-Planck equation above: see [35, 36] for useful introductions and discussion. However, the densities we are concerned with are densities on macroscopic states *𝒪* ⊂ *X* that ensure the microscopic states they enslave are far from thermodynamic equilibrium. It is these macroscopic states that are characteristic of biological agents. See [37, 38] for useful treatments in the setting of Darwinian dynamics. Having introduced the notion of entropy under ergodic assumptions, we next consider the implications of ergodicity for the flow or motion of agents through their state-space.

### 2.3. Global Random Attractors

A useful perspective on ergodic agents is provided by the theory of random dynamical systems. A random dynamical system is a measure-theoretic formulation of the solutions to stochastic differential equations like (3). It consists of a base flow (caused by random fluctuations) and a cocycle dynamical system (caused by flow). Ergodicity means the external states constitute a random invariant set $𝒜(\tilde{\omega })\subset X$ known as a *pullback* or *global random attractor* [19]. A random attractor can be regarded as the set to which a system evolves after a long period of time (or more precisely the pullback limit, after evolving the system from the distant past to the present: the pullback limit is required because random fluctuations make the system nonautonomous). In the limit of no random fluctuations, random attractors coincide with the definition of a deterministic attractor; as the minimal compact invariant set that attracts all deterministic bounded sets. Crucially, random attractors are compact subsets of state-space that are bounded by deterministic sets. Technically speaking, if the base flow is ergodic and $p(𝒜(\tilde{\omega })\subset 𝒪)>0$ then $𝒜(\tilde{\omega })=\Omega_{𝒪}(\tilde{\omega })$, almost surely [39]. Put simply, this means that if the random attractor falls within a bounded deterministic set *𝒪* ⊂ *X*, then it constitutes an omega limit set $\Omega_{𝒪}(\tilde{\omega })$. These are the states visited after a sufficiently long period, starting anywhere in *𝒪* ⊂ *X*. In short, if agents are random dynamical systems that spend their time within *𝒪* ⊂ X, then they have (are) random attractors. 

This existence of random attractors is remarkable because, in the absence of self-organising flow, the fluctuation theorem says they should not exist [40]. The fluctuation theorem generalises the second law of thermodynamics and states that the probability of a system's entropy decreasing vanishes exponentially with time. Put simply, random fluctuations disperse states, so that they leave any bounded set with probability one. See [41] and Appendix A, which show that in the absence of flow

(6) $$\dot{ℋ}\left(X\;|\;m\right)=\int_{X}\frac{\nabla p\cdot \Gamma \cdot \nabla p}{p\left(\tilde{x}\;|\;m\right)}d\tilde{x}\geq 0.$$

This says that random fluctuations increase entropy production in proportion to their amplitude and the roughness ∇*p* · ∇*p* of the ensemble density. In the absence of flow, the entropy increases until the density has dispersed and its gradients have been smoothed away. One can think of entropy as the volume or Lebesgue measure $\lambda (𝒜(\tilde{\omega }))$ of the attracting set: attractors with a small volume concentrate probability mass and reduce average surprise. One can see this heuristically by pretending that all the states within the attractor are visited with equal probability, so that $p(\tilde{x}|m)=1/\lambda :\tilde{x}\in 𝒜(\tilde{\omega })$. Under this assumption, one can see from (5) that *ℋ*(*X* | *m*) = ln⁡*λ* and that entropy increases with volume (and does so more acutely for small volumes). A low entropy means that a small number of states have a high probability of being occupied while the remainder have a low probability. This means that agents with well-defined characteristics have attractors with small measure and an ergodic density with low entropy. The implication here is that agents must counter the dispersive effects of random fluctuations to maintain a high ergodic density over the states *𝒪* ⊂ *X* they can occupy. It is important not to confuse the measure of an attracting set $\lambda (𝒜(\tilde{\omega }))$ with its topological complexity (although, strictly speaking, random attractors are a metric concept not topological). An attractor can have a small measure and yet have a complicated and space-filling shape. Indeed, one might suppose that complex agents (like us) have very complicated random attractors that support diverse and itinerant trajectories; like driving a car within a small margin of error.

### 2.4. Autopoiesis and Attracting Sets

The formulation of agents as ergodic random dynamical systems has a simple implication: it requires their flow to induce attractors and counter the dispersion of states by random fluctuations. In the absence of this flow, agents would encounter phase-transitions where macroscopic states collapse, exposing their microscopic states to thermodynamic equilibrium. But how do these flows arise? The basic premise, upon which the rest of this paper builds, is that these attractors are autopoietic [42] or self-creating (from the Greek: *auto* (**αυτ*ό*) for self- and *poiesis* (*π*ο*ί*ησις**) for creation). More formally, they arise from the minimisation of entropy with respect to action,

(7) $$a^{*}=\underset{a}{\arg\;\min}\;ℋ\left(X\;|\;m\right).$$

Action is the key to creating low entropy densities (resp., low measure attractors), because action determines flow and flow determines the ergodic density (resp., random attractor). This density is the eigensolution *ℰ*(Λ(**f**, Γ)) of the Fokker-Planck operator, which depends on the policy through the deterministic part of action and the amplitude of random fluctuations through the fluctuating part. This means action plays a dual role in controlling flow to attractive states and suppressing random fluctuations. Equation (6) shows that increasing the amplitude of controlled fluctuations increases the rate of entropy production, because $\partial_{\Gamma }\dot{ℋ}(X|m)>0$. This means the fluctuating part of action *ω*_*u*_ can minimise entropy production by suppressing the difference ${\tilde{\omega }}_{x}=\dot{\tilde{x}}-f\left(\tilde{x},u\right)={\tilde{\omega }}_{a}+\partial_{u}f\cdot \omega_{u}+\cdots$ between the flow experienced and that expected under the policy. This entails countering unexpected or random deviations from the policy to ensure an autopoietic flow (cf. a ship that maintains its bearing in the face of fluctuating currents and tides). In the absence of fluctuations, flow becomes deterministic and the random attractor becomes a deterministic attractor in the conventional sense (however, it is unlikely that action will have sufficient degrees of freedom to suppress controlled fluctuations completely). Note that for action to suppress random fluctuations about the expected flow (the policy) the agent must have a policy. We will address the emergence and optimisation of policies in the next section. At present, all we are saying is that action must minimise entropy and, implicitly, the measure of an agent's random attractor.

### 2.5. Summary

In summary, the ergodic or ensemble perspective reduces questions about adaptive behaviour to understanding how motion through state-space minimises surprise and its long-term average (conditional entropy). Action ensures motion conforms to an autopoietic flow or policy, given the agent and its current state. This policy induces a random invariant set $𝒜(\tilde{\omega })\subset 𝒪$ for each class of agent or species, which can be regarded as a probabilistic definition of the agent. This perspective highlights the central role played by the policy: it provides a reference that allows action to counter random fluctuations and violate the fluctuation theorem. In conclusion, the ergodic densities (resp. global random attractors) implied by the existence of biological agents are the stationary solutions to an autopoietic minimisation of their conditional entropy (resp. measure). In the next section, we consider what this implies for the functional anatomy of agents.

## 3. The Free Energy Formulation

In this section, we introduce the free energy principle as a means of minimising the conditional entropy of an agent's states through action. As noted above, these states and their entropy are hidden from the agent and can only be accessed through sensory states. This means that action cannot minimise the entropy of hidden states directly. However, it can do so indirectly by minimising the entropy of sensory states,

(8) $$a^{*}=\underset{a}{\arg\;\min\;}ℋ\left(X\;|\;m\right)=\underset{a}{\arg\;\min}\;ℋ\left(S\;|\;m\right).$$

This equivalence follows from two assumptions: there is a diffeomorphic mapping between hidden and sensory states and that Jacobian of this mapping (i.e., the sensitivity of sensory signals to their causes) is constant over the range of hidden states encountered (see Appendix B). Crucially, because sensory entropy is the long-term average of sensory surprise, the extremal condition above requires action to minimise the path integral of sensory surprise. This means (by the fundamental lemma of variational calculus) for *t* ∈ [0, *T*]

(9) $$\begin{array}{cc} \delta_{a}ℋ\left(S\;|\;m\right) & =0\Leftrightarrow \partial_{a(t)}ℒ\left(\tilde{s}\left(t\right)\right)=0\Leftrightarrow a{\left(t\right)}^{*}\\ & =\underset{a\left(t\right)}{\arg\;\min\;}ℒ\left(\tilde{s}\left(t\right)\right)\\ ℋ\left(S\;|\;m\right) & =\frac{1}{T}\overset{T}{\underset{0}{\int }}dtℒ\left(\tilde{s}\left(t\right)\right)\\ ℒ\left(\tilde{s}\left(t\right)\right) & =-\ln p\left(\tilde{s}\left(t\right)\;|\;m\right). \end{array}$$

Equation (9) says that it is sufficient for action to minimise sensory surprise to minimise the entropy of sensations (or at least find a local minimum). This is sensible because action should counter surprising deviations from the expected flow of states. However, there is a problem; agents cannot evaluate sensory surprise $ℒ(\tilde{s}(t))$ explicitly, because this would involve integrating $p(\tilde{s},\tilde{x},\theta |m)$ over hidden states and parameters or causes: $\vartheta =(\tilde{x},\theta )$. This is where the free energy comes in.

Free energy is a functional of sensory and internal states that upper bounds sensory surprise and can be minimised through action (cf. (2)). Effectively, free energy allows agents to finesse a generally intractable integration problem (evaluating surprise) by reformulating it as an optimisation problem. This well-known device was introduced by Feynman [43] and has been exploited extensively in machine learning and statistics [44–46]. The requisite free energy bound is created by adding a nonnegative Kullback-Leibler divergence or cross-entropy term [47] to surprise:

(10) $$\begin{array}{cc} ℱ\left(t\right) & =ℒ\left(\tilde{s}\left(t\right)\right)+D_{\text{KL}}\left(q\left(\vartheta \right)||\;p\left(\vartheta \;|\;\tilde{s},m\right)\right)\\ & ={\langle \ln q(\vartheta )\rangle }_{q}-{\langle \ln p\left(\tilde{s},\vartheta \;|\;m\right)\rangle }_{q}. \end{array}$$

This divergence is induced by a recognition density $q(\vartheta ):=q(\vartheta |\tilde{\mu })$ on the hidden causes of sensory states. This density is associated with the agent's internal states $\tilde{\mu }(t)$ that play the role of sufficient statistics; for example, the mean or expectation of hidden causes. Free energy $ℱ(\tilde{s},\tilde{\mu })\in \mathbb{R}$ can be evaluated because it is a functional of internal states and a generative model $p(\tilde{s},\vartheta |m)$ entailed by the agent. This can be seen from second equality, which expresses free energy in terms of the negentropy of *q*(*ϑ*) and the expected value of $\ln p(\tilde{s},\vartheta |m)$. 

To ensure action minimises surprise, the free energy must be minimised with respect the internal variables that encode the recognition density (to ensure the free energy is a tight bound on surprise). This is effectively perception because the cross-entropy term in (10) is non-negative, with equality when $q(\vartheta |\tilde{\mu })=p(\vartheta |\tilde{s},m)$ is the true conditional density. In short, optimising the recognition density makes it an approximate conditional density on the causes of sensory states. This is the basis of perceptual inference and learning as articulated by the Bayesian brain hypothesis [10, 13, 48–52]. We can now formulate action (9) in terms of a dual minimisation of free energy (see (2) and Figure 1).

(11) $$\begin{array}{c} a^{*}=\underset{a}{\arg\;\min\;}ℱ\left(\tilde{s},\tilde{\mu }\right),\\ {\tilde{\mu }}^{*}=\underset{\tilde{\mu }}{\arg\;\min}\;ℱ\left(\tilde{s},\tilde{\mu }\right). \end{array}$$

Action minimises free energy through changing the generalised motion of hidden states. In essence, it ensures that the trajectory of sensory states conform to the agents conditional beliefs encoded by internal states. Note that action is fundamentally different from a policy in optimal control and reinforcement-learning. Action is not a deterministic function of hidden states and is sensitive to random fluctuation causing sensory states. This means, unlike an optimal policy, it can suppress surprises by countering unexpected fluctuations in sensory states: although optimal control schemes can recover from perturbations, they cannot cancel them actively. However, as we will see below, optimal policies play a key role providing in prior constraints on the flow of hidden states that action tries to disclose.

### 3.1. Active Inference and Generalised Filtering

In what follows, we will assume that the minimisation of free energy with respect to action and internal states (11) conforms to a generalised gradient descent,

(12) $$\begin{array}{c} \dot{a}=-\partial_{a}ℱ\left(\tilde{s},\tilde{\mu }\right),\\ \dot{\tilde{\mu }}=𝒟\tilde{\mu }-\partial_{\tilde{\mu }}ℱ\left(\tilde{s},\tilde{\mu }\right). \end{array}$$

These coupled differential equations describe action and perception respectively. The first just says that action suppresses free energy. The second is known as generalised filtering [53] and has the same form as Bayesian (e.g., Kalman-Bucy) filtering, used in time series analysis. The first term is a prediction based upon the differential operator *𝒟* that returns the generalised motion of internal states encoding conditional predictions. The second term is usually expressed as a mixture of *prediction errors* that ensures the internal states (sufficient statistics) are updated in a Bayes-optimal fashion (see below). The differential equations above are coupled because sensory states depend upon action, which depends upon perception through the conditional predictions. This circular dependency leads to a sampling of sensory input that is both predicted and predictable, thereby minimising free energy and surprise. This is known as active inference.

In generalised filtering, one treats hidden parameters as hidden states that change very slowly: the ensuing generalised descent can then be written as a second-order differential equation: ${\ddot{\mu }}_{\theta }=-\partial_{\theta }ℱ-\kappa \mu_{\theta }^{\prime }$, where *κ* is the (high) prior precision on changes in hidden parameters. See [53] for details. In neurobiological formulations of free energy minimisation, internal states generally correspond to conditional expectations about hidden states and parameters, which are associated with neuronal activity and connections strengths, respectively. In this setting, optimising the conditional expectations about hidden states (neuronal activity) corresponds to *perceptual inference* while optimising conditional expectations about hidden parameters (neuronal plasticity) corresponds to *perceptual learning*.

Equation (12) describes the dynamics of action and internal states, whose particular form depends upon the generative model of the world. We will assume this model has the following form:

(13) $$\begin{array}{c} \dot{\tilde{x}}=f\left(\tilde{x},\theta \right)+{\tilde{\omega }}_{x},\\ \tilde{s}\left(t\right)=g\left(\tilde{x},\theta \right)+{\tilde{\omega }}_{s}. \end{array}$$

As in the previous section, (*f*, *g*) are nonlinear functions of hidden states that generate sensory states; however, these are distinct from the real equations of motion and sensory mappings (**f**, **g**) that depend on action. The generative model does not include action, because action is not a hidden state. Random fluctuations (*ω*_*s*_, *ω*_*x*_) play the role of sensory noise and induce uncertainty about the motion of hidden states. Hidden states are abstract quantities (like the motion of an object in the field of view) that the agent uses to explain or predict sensations. Gaussian assumptions about the random fluctuations in (13) furnish a probabilistic generative model of sensory states $p(\tilde{s},\vartheta |m)$ that is necessary to evaluate free energy. See [53] for a full description of generalised filtering in the context of hierarchical dynamic models. For simplicity, we have assumed that state-space associated with the generative model is the same as the hidden state-space in the world. However, this is not necessary, because exchanges with the environment are mediated through sensory states and action.

Given the form of the generative model (13) and an assumed (Gaussian) form for the recognition density, we can now write down the differential equations (12) describing the dynamics of internal states in terms of (precision-weighted) prediction errors $\left({\tilde{\varepsilon }}_{s},{\tilde{\varepsilon }}_{x}\right)$ on sensory states and the predicted motion of hidden states, where (ignoring some second-order terms and using $\tilde{g}:=g(\tilde{x},\theta )$)

(14) $$\begin{array}{cc} \dot{\tilde{\mu }} & =𝒟\tilde{\mu }+\partial_{\tilde{\mu }}\tilde{g}\cdot {\tilde{\varepsilon }}_{s}+\partial_{\tilde{\mu }}\tilde{f}\cdot {\tilde{\varepsilon }}_{x}-{𝒟}^{T}{\tilde{\varepsilon }}_{x},\\ {\tilde{\varepsilon }}_{s} & ={\tilde{\Pi }}_{s}\left(\tilde{s}-\tilde{g}\right),\\ {\tilde{\varepsilon }}_{x} & ={\tilde{\Pi }}_{x}\left(𝒟\tilde{\mu }-\tilde{f}\right). \end{array}$$

The (inverse) amplitude of generalised random fluctuations are encoded by their precision $\left({\tilde{\Pi }}_{s},{\tilde{\Pi }}_{x}\right)$, which we assume to be fixed in this paper. This particular free energy minimisation scheme is known as *generalised predictive coding *and has become a useful metaphor for neuronal message passing in the brain: see also [12]. The simplicity of this scheme stems from the assumed Gaussian form of the recognition density. This means the internal states or sufficient statistics can be reduced to conditional expectations (see Appendix C). In neural network terms, (14) says that error-units receive predictions while prediction-units are driven by prediction errors. In neurobiological implementations of this scheme, the sources of prediction errors are usually thought to be superficial pyramidal cells while predictions are conveyed from deep pyramidal cells to superficial pyramidal cells encoding prediction error [54]. 

Because action can only affect the free energy by changing sensory states, it can only affect sensory prediction errors. From (13), we have

(15) $$\dot{a}=-\partial_{a}\tilde{s}\cdot {\tilde{\varepsilon }}_{s}.$$

In biologically plausible instances of active inference, the partial derivatives in (15) would have to be computed on the basis of a mapping from action to sensory consequences, which is usually quite simple; for example, activating an intrafusal muscle fibre elicits stretch receptor activity in the corresponding spindle: see [6] for discussion.

### 3.2. Summary

In summary, we can account for the unnatural persistence of self-organising biological systems in terms of action that counters the dispersion of their states by random fluctuations. This action minimises the entropy of their ergodic density by minimising a free energy bound on sensory surprise or self-information as each point in time. To ensure the free energy is a good proxy for surprise, internal states must also minimise free energy and implicitly represent hidden states. This minimisation rests upon a generative model, which furnishes conditional predictions that action can fulfil. These predictions rest of on equations of motion that constitute (empirical) priors [55] on the flow of hidden states in the world. In short, agents are equipped with a model of dynamics in their local environment and navigate that environment to minimise their surprise.

We can now associate the expected flow of the previous section with the empirical priors learned under the generative model: **f**(*x*, *u*) = *f*(*x*, *μ*_*θ*_). This rests upon the assumption that action eliminates (on average) the difference between the actual and predicted flow. This means the predicted flow corresponds to the policy. The policy **f**(*x*, *u*) = *f*(*x*, *μ*_*θ*_) is an *empirical* prior because it depends on conditional beliefs about hidden parameters encoded by *μ*_*θ*_. This is an important point because it means that the environment causes prior beliefs about motion (through parameter learning), while these beliefs cause the sampled environment. This circular causality is the basis of autopoietic flow and highlights the fact self-organisation rests on a reciprocal exchange between implicit beliefs about how an agent or system will behave and behavioural constraints that are learned by behaving. Minimising free energy ensures that the beliefs and constraints are consistent and enables the agent to create its own environment. In this view, perceptual inference becomes truly embodied or situated and is an integral part of sustainable interactions with the environment. The previous section suggested that action was the key to understanding self-organised behaviour. This section suggests that action depends on a policy or empirical priors over flow. In what follows, we consider the nature of this flow and its specifications.

## 4. Policies and Value

The previous section established differential equations that correspond to action and perception under a model of how hidden states evolve. These equations are based on the assumption that agents suppress (a bound on) surprise and, implicitly, the entropy of their ergodic density. We now consider optimising the model per se, in terms of formal priors on flow. These correspond to the form of the equation of motions in (13). In particular, we will consider constraints encoded by a (cost) function $c(\tilde{x})\subset m$ over hidden states. The existence of autopoietic flow is not mysterious, in the sense that agents who do not have a random attractor cannot exist. In other words, every agent (phenotype) can be regarded as a solution to the Fokker-Planck equation, whose policy is compatible with the biophysics of its environmental niche. One might conjecture that each solution (random attractor) corresponds to a different species, and that there may be a limited number of solutions as evidenced by convergent evolution [17]. This section considers the policies that underwrite these solutions and introduces the notion of *value* in terms of the Helmholtz decomposition. In brief, we will see that flow determines value, where value is negative surprise.

We start with the well-known decomposition of flow into curl- and divergence-free components (strictly speaking, the first term is only curl-free when $\Gamma \left(\tilde{x}\right)=\gamma \left(\tilde{x}\right)\cdot I$; that is, the diffusion tensor is isotropic. However, this does not affect the following arguments, which rest on the divergence-free component),

(16) f=Γ·∇V+∇×W.

This is the Helmholtz decomposition (also known as the fundamental theorem of vector calculus) and expresses any policy in terms of scalar $V(\tilde{x})$ and vector $W(\tilde{x})$ potentials that prescribe irrotational (curl-free) Γ · ∇*V* and solenoidal (divergence-free) ∇×*W* flow. An important decomposition described in [37, 56], formulates the divergence-free part in terms of an antisymmetric matrix, $Q(\tilde{x})=-Q{\left(\tilde{x}\right)}^{T}$ and the scalar potential, which we will call *value*, such that

(17) $$\begin{array}{c} f=\left(\Gamma +Q\right)\nabla V\Rightarrow \\ \nabla \times W=Q\nabla V. \end{array}$$

Using this (*standard form*) decomposition [57], it is fairly easy to show that $p(\tilde{x}|m)=\exp(V(\tilde{x}))$ is the equilibrium solution to the Fokker-Planck equation (4):

(18) $$\begin{array}{cc} p & =\exp\left(V\right)\Rightarrow \nabla p=p\nabla V\Rightarrow \\ \Lambda p & =\nabla \cdot \Gamma \nabla p-\nabla \cdot \left(fp\right)\\ & =-p\left(\nabla \cdot \left(Q\nabla V\right)+\left(Q\nabla V\right)\cdot \nabla V\right)=0. \end{array}$$

Equation (18) uses the fact that the divergence-free component is orthogonal to ∇*V* (see Appendix D). This straightforward but fundamental result means that the flow of any ergodic random dynamical system can be expressed in terms of orthogonal curl- and divergence-free components, where the (dissipative) curl-free part increases value while the (conservative) divergence-free part follows isoprobability contours and does not change value. Crucially, under this decomposition value is simply negative surprise: $\ln p(\tilde{x}|m)=V(\tilde{x})=-ℒ(\tilde{x}|m)$. It is easy to show that surprise (or value) is a Lyapunov function for the policy

(19) $$\begin{array}{cc} \dot{V}\left(x\left(t\right)\right) & =\nabla V\cdot f=\nabla V\cdot \Gamma \cdot \nabla V+\nabla V\cdot \nabla \times W\\ & =\nabla V\cdot \Gamma \cdot \nabla V\geq 0. \end{array}$$

Lyapunov functions always decrease (or increase) with time and are used to establish the stability of fixed points in deterministic dynamical systems. This means every policy (expected flow) reduces surprise as a function of time. In other words, it must direct flow towards states that are more probable (and have a greater sojourn time). This is just a formal statement of the fact that ergodic systems must, on average, continuously suppress surprise, to offset the dispersive effect of random fluctuations. Ao reviews the importance and generality of the decomposition in (17) and how it provides a unifying perspective on evolutionary and statistical dynamics [38]: this decomposition shows that fluctuations in Darwinian dynamics imply the existence of canonical distributions of the Boltzmann-Gibbs type. Furthermore, it demonstrates the second law of thermodynamics, without detailed balance. In particular, the dynamical (divergence-free) component responsible for breaking detailed balance does not contribute to changes in entropy. In short, (17) represents “a simple starting point for statistical mechanics and thermodynamics and is consistent with conservative dynamics that dominates the physical sciences” [58]. The generality of this formulation can be appreciated by considering two extreme cases of flow that emphasise the curl and divergence-free components, respectively.

### 4.1. Conservative (Divergence-Free) Flow

When the random fluctuations are negligible (i.e., Γ → 0), irrotational (curl-free) flow Γ · ∇*V* = 0 disappears and we are left with divergence-free flow that describes conservative dynamics (e.g., classical mechanics). These flows would be appropriate for massive bodies with virtually no random fluctuations in their motion. A simple example would be the Newtonian mechanics that result from a Lagrangian (surprise) and antisymmetric matrix,

(20) $$\begin{array}{c} ℒ\left(\tilde{x}\right)=\varphi \left(x\right)+\frac{1}{2}x^{\prime 2}\\ Q=\left[\begin{array}{cc} 0 & -1\\ 1 & 0 \end{array}\right]\Rightarrow f=-Q\nabla ℒ=\left[\begin{array}{c} \dot{x}\\ {\dot{x}}^{\prime } \end{array}\right]=\left[\begin{array}{c} x^{\prime }\\ -\nabla \varphi \end{array}\right]. \end{array}$$

This describes the motion of a unit mass in a potential field *φ*(*x*), where the Lagrangian comprises potential and kinetic terms. Things get more interesting when we consider random fluctuations in the velocity,

(21) $$\begin{array}{cc} \Gamma & =\left[\begin{array}{cc} 0 & 0\\ 0 & \gamma \end{array}\right]\Rightarrow \\ f & =-\left(\Gamma +Q\right)\nabla ℒ=\left[\begin{array}{c} x^{\prime }\\ -\nabla \varphi -\gamma x^{\prime } \end{array}\right]. \end{array}$$

This introduces a motion-dependent reduction in the motion of velocity (acceleration) that corresponds to friction. Note that friction is an emergent property of random fluctuations in velocity (and nothing more). A more thorough treatment of the relationship between the diffusion due to random fluctuations and friction can be found in [57], using the generalised Einstein relation. Consider now systems in which random fluctuations dominate and the conservative (divergence-free) flow can be ignored.

### 4.2. Dissipative (Curl-Free) Flow and Detailed Balance

Here, irrotational (curl-free) flow dominates and the dynamics have *detailed balance*, which means that flow can be expressed as an ascent on a scalar (value) potential: *f* = Γ · ∇*V* = −Γ · ∇*ℒ*. Crucially, because there is effectively no conservative flow, the ergodic density concentrates around the maximum of value (or minimum of surprise), which (in the deterministic limit) induces a fixed point attractor. Curl-free polices are introduced here, because of their central role in optimal control and decision (game) theory: in the next section, we will consider curl-free policies that are specified in terms of value-functions, $V(\tilde{x})$. These range from reinforcement-learning heuristics in psychology to more formal optimal control theory treatments. However, one should note that these approaches are incomplete in the sense they do not specify generic policies: a complete specification of flow would require the vector potential $W(\tilde{x})$ or, equivalently, the anti-symmetric matrix, $Q(\tilde{x})$. This means that it is not sufficient to know (or learn) the value of a state to specify a policy explicitly, unless the environment permits curl-free policies with detailed balance (i.e., with no classical or conservative dynamics). 

Ergodic densities under detailed balance are closely connected to *quantal response equilibria* (QRE) in economics and game theory. QRE are game-theoretical formulations that provide an alternative to Nash equilibria [18]. QRE do not require perfect rationality; players are assumed to make normally distributed errors in their predicted payoff. In the limit of no errors, QRE predict unique Nash equilibria. From the point of view of game theory, the interesting questions pertain to different equilibria prescribed by the policy or state-transitions. These equilibria are analogous to the solutions of the Fokker-Planck equation above, where $V(\tilde{x})$ is called *attraction* and Γ ∈ ℝ^+^ is temperature or inverse sensitivity [9, 59]. In this context, the ergodic density $p(\tilde{x}|m)\;=\exp(-ℒ)$ prescribes optimal states or choices probabilistically, in terms of value, where *V* = −*ℒ*. This prescription is closely related to softmax or logit discrete choice models [60], which are the most common specification of QRE. In economics, optimal state-transitions lead to equilibria that maximise value or expected utility. These are low-entropy densities with probability mass on states with high utility. We purse this theme in below, in the context of optimal control theory and reinforcement-learning.

### 4.3. Summary

In this section, we have seen that a policy or empirical priors on flow (specified by conditional beliefs about the parameters of equations of motion) can be decomposed into curl and divergence-free components, specified in terms of a value-function and antisymmetric matrix that determines conservative flows of the sort seen in classical mechanics. Crucially, this value-function is just negative surprise and defines the ergodic (invariant) probability density over hidden states. However, we have no idea about where the policy comes from. All we know is that it furnishes a solution to the Fokker-Planck equation; an idiocentric description of an agent's exchange with the environment. The remainder of this paper will be concerned with how policies are specified and how they are instantiated in terms of value-functions.

Evolutionary theory [61, 62] suggests that species (random attractors) do not arise *de novo* but evolve through natural selection (e.g., by punctuated equilibria or phyletic gradualism; [63, 64]). We take this to imply that policies are heritable and can be encoded (epigenetically) in terms of value or cost-functions. We will assume the agents are equipped with a cost-function that labels states as attractive or not

(22) $$\begin{array}{cc} & c\left(x\;|\;m\right)\leq 0:\;x\in 𝒜=\underset{\tilde{\omega }\in \Omega }{⋂}𝒜\left(\tilde{\omega }\right),\\ & c\left(x\;|\;m\right)>0:\;x\notin 𝒜. \end{array}$$

Technically, cost indicates whether each state is in a *kernel* or the set of *fixed points* of a random attractor [65]. In the deterministic limit Γ → 0 this kernel reduces to an attractor in the usual sense. From now on, we will use *𝒜* ⊂ *𝒪* to mean the kernel of a random attractor or an attractor in the deterministic sense. The introduction of cost allows us to connect attractors in dynamical systems with attractive states in reinforcement-learning and optimal control. Informally, cost labels states as either attractive (e.g. sated) or costly (e.g., thirsty). The cost-function could also be regarded as a characteristic function that indicates whether the current state is characteristic of the class the agent belongs to. This labelling is sufficient to prescribe policies that assure equilibrium solutions, as evidenced by the existence of evolved agents. This question is how? We will begin by considering control theory.

## 5. Optimal (Fixed Point) Control and Reinforcement-Learning

In this section, we look at policies and value from the point of view of optimal control theory and reinforcement-learning. In the previous section, value was considered to arise from a decomposition of flow into curl, and divergence-free parts. In that setting, value simply reports the surprise that a state is occupied. In other words, value is an attribute of the policy. Optimal control theory turns this around and assumes that the policy is an attribute of value. This enables policies to be specified by value, via cost-functions. In this section, we will consider optimal control theory as optimising policies (flows), whose attracting fixed-points are specified by cost-functions. Crucially, because optimal control policies do not specify divergence-free flow, they can only specify policies with attracting fixed points (the maxima of the value function). In the next section, we turn to generalised policies that exploit divergence-free flow to support itinerant policies. We will persist with continuous time formulations in this section and provide discrete time versions of the main results in the appendices.

### 5.1. Optimal Control Theory

In optimal control theory and its ethological variants (i.e., reinforcement-learning), adaptive behaviour is formulated in terms of how agents navigate state-space to access sparse rewards and avoid costly regimes. The aim is to find a (proximal) policy that attains long-term (distal) rewards. In terms of the previous section, a policy *f* = Γ · ∇*V* + ∇×*W* was specified via the scalar potential or value $V(\tilde{x})$ also known as (negative) *cost-to-go*. In optimal control theory, value is defined as the expected path-integral of cost. More formally, the cost-to-go of ${\tilde{x}}_{0}\in X$ is the cost expected over future times *t* ∈ [*t*_0_, *∞*], starting with a point density $p(\tilde{x},t_{0}|m)=\delta ({\tilde{x}}_{0})$, which evolves according to (3) (see Appendix E),

(23) $$\begin{array}{cc} V\left({\tilde{x}}_{0}\right) & =-\overset{\infty }{\underset{t_{0}}{\int }}\overset{}{\underset{}{\int }}c\left(\tilde{x}\right)p\left(\tilde{x},t\;|\;m\right)dx\;dt\Rightarrow \\ c\left(\tilde{x}\right) & =f\cdot \nabla V\left(\tilde{x}\right)+\nabla \cdot \Gamma \cdot \nabla V\left(\tilde{x}\right). \end{array}$$

Or in the deterministic limit Γ → 0

(24) $$\begin{array}{cc} V\left({\tilde{x}}_{0}\right) & =-\overset{\infty }{\underset{t_{0}}{\int }}c\left(\tilde{x}\left(t\right)\right)dt\Rightarrow \\ c\left(\tilde{x}\right) & =f\cdot \nabla V\left(\tilde{x}\right)=\dot{V}\left(\tilde{x}\left(t\right)\right). \end{array}$$

This definition of value as an expected path-integral of cost (first line) allows cost to be expressed as a function of value (second line). It says that cost is the expected increase in value. This may sound counterintuitive but makes sense if one considers a reward now means less in the future (i.e., a decrease in the value of the next state). Crucially, (24) shows that the maxima of the ergodic density can only exist where cost is zero or less (cf. (22)): at a maximum of $p(\tilde{x}|m)=\exp(V)$ we have the following:

(25) $$\begin{array}{c} \nabla V\left(\tilde{x}\right)=0\\ \nabla \cdot \Gamma \cdot \nabla V\left(\tilde{x}\right)\leq 0 \end{array}\}\Rightarrow c\left(\tilde{x}\right)\leq 0,$$

with $c(\tilde{x})=0$ in the deterministic limit. Put simply, costly regions induce value gradients that guide flow towards points where there is no cost (i.e., no gradients). In this sense, value is sometimes called a navigation function. This means that, in principle, we have a way to prescribe ergodic densities with maxima (attracting fixed-points) that are specified with a cost-function. Equation (23) shows that the cost-function can be derived easily, given the policy and implicit value-function. However, to specify a policy with cost, we have to derive the flow from the cost-function. This entails associating a unique flow with the value-function and solving (24) for value: this association is necessary because optimal control does not specify the divergence-free part of the policy. Solving (24) is the difficult problem optimal control and value-learning deal with.

Let optimal control be denoted by $\pi (\tilde{x})$, where optimal control maximises the ascent of the value-function

(26) $$\pi \left(\tilde{x}\right)=\underset{u}{\arg\;\max}\;f\left(\tilde{x},u\right)\cdot \nabla V\left(\tilde{x}\right).$$

This extremal condition associates a unique (optimal) flow with every value-function such that value can be expressed in terms of cost using  (24)

(27) $$\underset{u}{\max}\;f\left(\tilde{x},u\right)\cdot \nabla V\left(\tilde{x}\right)=f\left(\tilde{x},\pi \right)\cdot \nabla V\left(\tilde{x}\right)=c\left(\tilde{x}\right).$$

This is the celebrated Hamilton-Jacobi-Bellman (HJB) equation. More general forms are provided in Appendix F for the interested reader. The basic problem, posed by the solution of the HJB equation for value, is that the value-function depends on optimal control, so that future cost can be evaluated. However, optimal control depends on the value-function. This circular dependency can only be resolved by solving the self-consistent equations above, also known as the dynamic programming recurrence. This is the *raison d'être *for value-learning.

In engineering, planning, and control problems, the HJB equation is usually solved by backwards induction (staring at the desired fixed-point and working backwards). However, this is not an ethological option for agents that have to learn the value-function online. An alternative solution exploits the fact that the expected increase in the value of the current state is cost. This leads to a straightforward value-learning scheme

(28) $$V\left(x\left(t\right)\right)⟵V\left(x\left(t\right)\right)+\delta :\delta =\dot{V}\left(x\left(t\right)\right)-c\left(x\left(t\right)\right).$$

Such that, at convergence, the value-function satisfies the deterministic limit of (24), at least for the states visited,

(29) $$\delta \left(t\right)\to 0\Rightarrow c\left(x\right)=\dot{V}\left(x\left(t\right)\right)=f\cdot \nabla V\left(x\right).$$

Heuristically, this scheme erodes the value landscape, creating gradients that ensure flow through costly regions of state-space. The only points that are exempt from this erosion are maxima with zero flow and cost (or negative cost in the presence of fluctuations). These are the fixed-points of the attracting set prescribed by the cost-function. 

In (28), *δ*(*t*) reports the difference between the cost predicted $\dot{V}(x(t))$ and observed *c*(*x*(*t*)). This is the negative cost or reward prediction error. There is a vast literature on reinforcement-learning schemes that solve the discrete time version of the HJB equation; either by backwards induction (model-based schemes) or by using reward prediction error (model-free schemes). Model-free schemes use a discrete time version of (28) using the Robbins-Monro algorithm; [66, 67].  Appendix G provides a brief survey of these schemes. Intuitively, they all involve increasing the value of the last state in proportion to the reward prediction error. This is the basis of temporal difference schemes [2] and Rescorla-Wagner [68] models of conditioning in psychology. See [5] for a comprehensive review. If the agent has no model of flow or state-transitions, similar schemes can be invoked to optimise the policy, (e.g., actor-critic schemes). A generalisation of value-learning, called Q-learning, considers a value or *quality Q* : *X* × *U* → ℝ on the joint space of states and control [69]. Q-learning does not need a model in the form of probabilistic transitions to optimise control, because the quality of an action is encoded explicitly. Perhaps the most important thing to come out of these modelling initiatives is that phasic dopamine discharges in the brain are a prime candidate for reporting reward prediction error [3, 70, 71]. In some cases theoretical predictions preempted empirical findings; for example, “in the absence of an expected reward, value system responses should show a decrease in activity at the time when the reward would normally be delivered” [72], which was confirmed subsequently [73].

### 5.2. Summary

**Figure 4:**
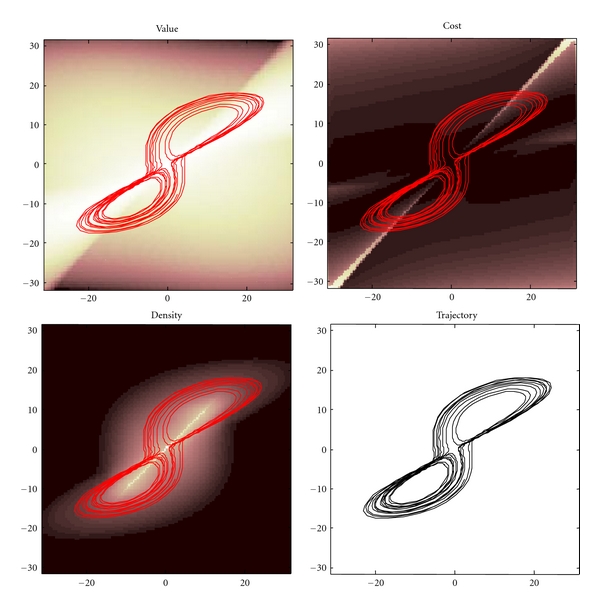
Value and cost functions of dynamical systems. This figure shows the value and cost functions of the Lorentz attractor used in the previous figures. These functions always exist for any global random attractor because value (negative surprise) is the log density of the eigensolution of the systems Fokker-Planck operator. This means, given any deterministic motion (flow) and the amplitude of random fluctuations (diffusion), we can compute the Fokker Planck operator Λ(*f*, Γ) and its eigensolution *p* = *ℰ*(Λ) and thereby define value *V* = ln⁡*p*. Having defined value, cost is just the expected rate of change of value, which is given by the deterministic flow and diffusion (see (23)). In this example, we computed the eigensolution or ergodic density using a discretisation of state-space into 96 bins over the ranges: [−32,  32]×[−32,  32]×[4,  64] and a diffusion tensor of Γ = (1/64) · *I*. The upper panels show the resulting value and (negative) cost functions for a slice through state-space at *x*_3_ = 24. Note how cost takes large values when the trajectory (red line) passes through large value gradients. The lower left panel shows the resulting ergodic density as a maximum intensity projection over the third state. A segment of the trajectory producing this density is shown on the lower right.

In summary, one can decompose any policy or expected flow into a part that is divergence-free and a part that increases value, where value is negative surprise or the log probability of a state being occupied. This means, given expected flow and the amplitude of random fluctuations about that flow, one can compute the ergodic density and associated value-function. Furthermore, if one defines surprise (negative value) of any state as the expected cost accumulated from that state, then it is straightforward to evaluate the cost-functions implied by any given policy. An example is given in Figure 4 using the Lorentz attractor in previous figures.

Using this definition of cost, reinforcement-learning and optimal control theory try to derive value from cost-functions by assuming controlled flow minimises accumulated cost. This involves solving self-consistent equations that entail these assumptions. The ensuing value-function guides flow to ensure cost is minimized under constraints on motion. In dynamical terms, this approach optimises a policy in terms of its scalar potential, whose maxima coincide with points in state-space where *c*(*x*) ≤ 0. However, despite its prominence in the neurosciences, optimal control theory is not very useful for understanding self-organisation in biological systems. For example, one could not specify the policy followed by the Lorentz attractor in Figure 4, because its dynamics do not have detailed balance. In other words, although one can derive a cost-function from any flow, one cannot specify any flow with a cost-function: to specify any given policy one would need the vector potentials (or anti-symmetric matrices) above.

Furthermore, optimal control schemes and related heuristics have several shortcomings: (i) they are notoriously slow, requiring hundreds if not thousands of trials to learn even the simplest value-function, (ii) value-learning based on stochastic iteration depends on the same random fluctuations that need to be suppressed to pursue the policy, (iii) optimal control theory says nothing about exploration of state-space, (iv) an exigent limitation of these schemes is that they only account for policies with stationary fixed-points (i.e., agents who would optimally do nothing). This means they cannot account for the itinerant and context-sensitive nature of real behaviour. To resolve these problems we now turn to generalised policies that include divergence-free flow and are constrained directly, as opposed to placing constraints on value functions.

## 6. Generalised (Itinerant) Policies

In the previous section, cost was used to specify policies or expected flow in terms of value-functions. However, policies with detailed balance (of the form *f* = Γ · ∇*V*) place severe constraints on the attractors they engender. In the deterministic limit, they can only prescribe attractors with a single fixed-point (per basin of attraction), which is a local maximum of value *V* : *X* → ℝ. This is not a useful description of real agents that exhibit itinerant dynamics with quasiperiodic and chaotic behaviour. In what follows, we consider policies in generalised coordinates of motion that do not have detailed balance (and exploit divergence-free flow); we will refer to these as generalised policies. These policies provide a rich repertoire of dynamics with attracting sets that can be specified directly by policies. In other words, we dispense with the assumptions of optimal control theory and consider the more general problem of how to specify attracting sets. In what follows, cost-functions are used to specify (unstable) fixed points for fairly generic flows. We will see how this leads to a dynamical formulation of policies and the emergence of optimal itinerancy. This section focuses on the basics and provides some simple examples, noting that there are many potential extensions; some of which have already been addressed in the application of dynamical systems theory to biological systems (see below). 

Generalised policies rest on the ensemble dynamics perspective: we start by considering how cost can restrict the probability mass of an ergodic density to a subset of state-space *𝒜* ⊂ X. The Fokker-Planck equation (3) provides a fundamental constraint on flow that must be satisfied when Λ*p* = 0 and

(30) $$\begin{array}{c} \nabla \cdot \Gamma \nabla p=\nabla \cdot \left(fp\right)=f\cdot \nabla p+p\nabla \cdot f\Rightarrow \\ p\left(\tilde{x}\;|\;m\right)=\frac{\nabla \cdot \Gamma \nabla p-f\cdot \nabla p}{\nabla \cdot f}. \end{array}$$

This straightforward result shows that as divergence ∇·*f* decreases, the sojourn time (i.e., the proportion of time a state is occupied) rises. This means divergence decreases value and increases surprise. This is intuitive, in that divergence represents the extent to which flow behaves like a source or a sink at a given point. Attractive or valuable points in state-space are sinks with low divergence. At the peaks of the ergodic density its gradient is zero and its curvature is negative:

(31) $$\begin{array}{c} p>0\\ \nabla p=0\\ \nabla \cdot \Gamma \cdot \nabla p<0 \end{array}\}\Rightarrow \nabla \cdot f<0.$$

This means divergence must be negative. This generalises an almost trivial result in deterministic systems: divergence is the sum of the flow's real Lyapunov exponents ∇·f=tr⁡(∂x˜f)=∑iRe(λi), where *λ*_*i*_ are the eigenvalues of the Jacobian $\partial_{\tilde{x}}f$. If divergence is negative, then all the Lyapunov exponents are negative, implying a stable fixed-point attractor. 

This provides a straightforward way of ensuring the peaks of the ergodic density lie in, and only in *𝒜* ⊂ *X*. This is assured if ∇·*f* < 0 when $\tilde{x}\in A$ and ∇·*f* ≥ 0 otherwise. We can exploit this using the equations of motion in  (21)

(32) $$\begin{array}{c} f\left(x,x^{\prime }\right)=\left[\begin{array}{c} x^{\prime }\\ c\left(x\right)\cdot x^{\prime }-\partial_{x}\varphi \end{array}\right]\Rightarrow \nabla \cdot f=c \end{array}.$$

This flow describes the classical (Newtonian) motion of a unit mass in a potential energy well *φ*(*x*), where cost plays the role of negative dissipation or friction. Crucially, under this policy or flow, divergence is cost. This means the associated ensemble density can only have maxima in regions of negative (*divergence-based*) cost. This provides a means to specify attractive regions *𝒜* ⊂ X by assigning them negative cost, which brings us back to (22),

(33) $$\begin{array}{c} c\left(x\right)\leq 0:\;x\in 𝒜,\\ c\left(x\right)>0:\;x\notin 𝒜. \end{array}$$

Put simply, this scheme ensures that agents are expelled from high-cost regions of state-space and get “stuck” in attractive regions. Equivalently, cost can be regarded as destroying unattractive fixed points at the minima of the potential landscape *φ*(*x*). It should be noted that negative divergence does not ensure an attractive fixed point; however, if divergence is sufficiently negative, the point will act as a sink and become, almost surely, part of the attracting set. We will see an example of this below. In summary, in attractive regions with low cost, flow slows down sufficiently to increase sojourn time and implicitly value. This is not dissimilar to Win-Stay, Lose-Shift strategies used to model optimal decisions in game theory [74]. We will now illustrate how divergence-based cost works using the mountain car problem.

### 6.1. The Mountain Car Problem

Here, we use active inference and a generative model based on (31) to solve a fairly difficult problem in optimal control based purely on the theoretical treatment above. Crucially, the agent that solves this problem has no prior information about constraints on its action and yet it can respond adaptively, when supplied with a cost-function, to find its goal almost immediately. Note that there is no value-learning because we use divergence-based cost, which does not require a value-function. Furthermore, this behaviour is resilient to perturbations, because the policy provides predictions, which are fulfilled by action.

**Figure 5:**
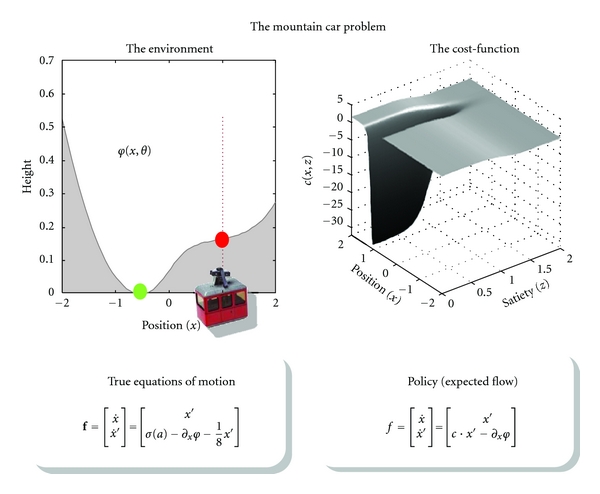
The mountain car problem. The upper left panel shows the landscape or potential energy function *φ*(*x*, *θ*), with a minimum at position, *x* = −0.5 (green dot) that exerts forces on the car. The car is shown at the target position at the top of the hill at *x* = 1 (red dot). The equations of motion of the car are shown below. Crucially, at *x* = 0 the force is unity and cannot be overcome by the agent, because a squashing function −1 ≤ *σ*(*a*) ≤ 1 is applied to action. This means the agent can only access the target by starting on the left hill to gain enough moment to carry it up the other side. The right panels show the cost function and empirical priors (model of flow) that constitute the agent. Cost is a function of position and a hidden (e.g., physiological) state that plays a role of satiety *c*(*x*, *z*) = (16 · exp⁡(−64(*x*−1)^2^) − 1)·(tanh(8(z − 1)) − 1) − 1. When satiety is high, cost is uniformly negative; *c*(*x*, *∞*) = −1. Conversely, when satiety is low cost becomes negative near, and only near, the target location; *c*(*x*, 0) = 1 − 32 · exp⁡(−64(*x* − 1)^2^). The equations of motion on the lower right are constructed to ensure that fixed points are only stable in regions of negative cost or divergence: see main text.

**Figure 6:**
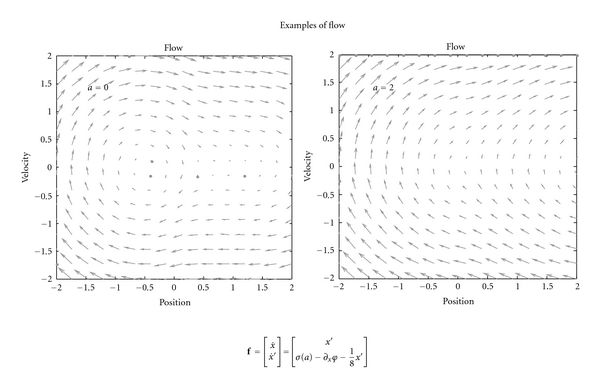
Examples of flow. This figure provides two examples of flow under two levels of action based on the equations of motion in the previous figure (the mountain car problem). These action-dependent flows provide a repertoire from which the agent has to compose a policy that conforms to its prior beliefs.

In the mountain car problem, one has to park a mountain car halfway up a hill. However, the car is not sufficiently powerful to ascend the hill directly. This means the only solution to problem is to back away from the parking location and then accelerate towards it, in the hope of acquiring sufficient momentum to access the target. The upper left panel of Figure 5 shows the landscape or potential energy function (with a minimum at position, *x* = −0.5) that exerts forces on the car. The car is shown at the target position at the top of the hill at *x* = 1 (red dot). The equations of motion of the car are shown below. Crucially, at *x* = 0 the force is unity and cannot be overcome by the agent, because a squashing function −1 ≤ *σ*(*a*) ≤ 1 is applied to action. The right panels show the agent's generative model in terms of the equations of motion in (13). These correspond to (31), where the cost-function is shown on the upper right. Here, the cost-function *c*(*x*, *z*) has an auxiliary parameter *z* ∈ ℝ that enables cost to be switched on or off. This can be thought of as satiety, such that when *z* = 0 cost is positive everywhere, except the target location (see figure legend for details). In this example, the sensory mapping and its assumed form were just $g(\tilde{x})=g(\tilde{x})=\tilde{x}$. Notice that the true equations of motion depend on action while the policy does not. This means the actual behaviour of the agent is selected from a family of flows by action.  Figure 6 shows two exemplar flows under different values for action. Under active inference, action tries to realise conditional beliefs that are specified by the policy or empirical priors on motion.

**Figure 7:**
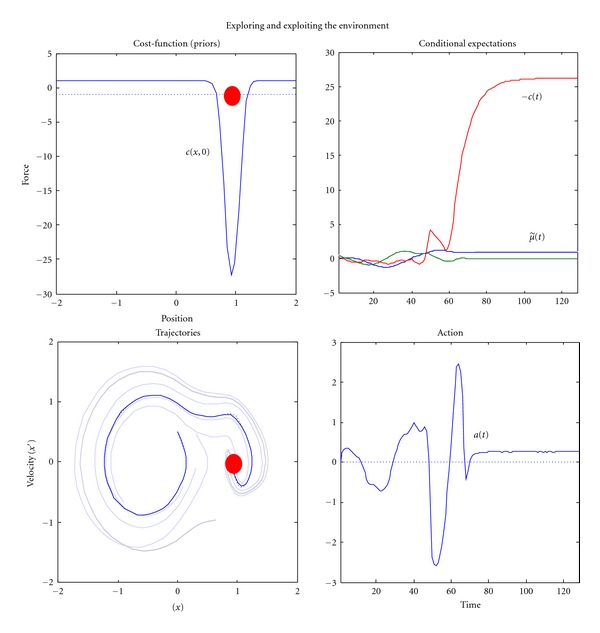
Active inference with generalised policies. This example shows how paradoxical but adaptive behaviour (moving away from a target to secure it later) emerges from simple priors on the motion of hidden states. These priors are encoded in a cost function *c*(*x*, 0) (upper left). The form of the agent's (generalised) policy ensures that divergence is positive or friction is negative in regions of positive cost, such that the car expects to go *faster*. The inferred hidden states (upper right: position in blue, velocity in green, and friction in red) show that the car explores its landscape until it encounters the target and friction increases dramatically to prevent it escaping (i.e., falling down the hill). The ensuing trajectory is shown in blue (lower left). The paler lines provide exemplar trajectories from other trials, with different starting positions. In the real world, friction is constant (one eighth). However, the car expects friction to change with position, enforcing exploration or exploitation. These expectations are fulfilled by action (lower right), which tries to minimise free energy.

Figure 7 shows how paradoxical but adaptive behaviour (moving away from a target to ensure it is secured later) emerges from this sort of generalised policy on the motion of hidden states, using *c*(*x*, 0). These simulations are the results of integrating (1) and (2) (see Appendix H for details). The inferred hidden states (upper right) show that the car explores its landscape until it encounters the target and negative cost or friction increases dramatically to prevent it escaping (i.e., falling down the hill). This ensuing trajectory is shown on the upper left panel. The paler lines provide exemplar trajectories from other trials, with different starting positions. In the real world, friction is constant (one eighth). However, the car expects friction to change with position, enforcing exploration or exploitation. These expectations are fulfilled by action (lower right).

It is important to appreciate what has and what has not been achieved in this simulation: using a single scalar cost-function of position we have been able to induce adaptive goal-directed behaviour without any value-learning or enforced exploration of the environment. This behaviour is elicited immediately without the need for repeated trials or exposures. Furthermore, the requisite exploration and exploitation is manifest as a direct consequence of the agent's priors, without the need for carefully calibrated stochastic terms during training. This sort of adaptive behaviour is reminiscent of foraging seen in insects and other biological agents; where the environment is explored under autonomous dynamics until some reward is encountered. In an experimental context, the above simulation could be regarded in terms of eliciting foot-shock escape behaviour [75]; in that the uniformly high cost can only be avoided by escaping to a low-cost location. It may seem implausible to specify behaviour in terms of cost that is generally high everywhere; however, one can see easily how drive states might correspond to high cost and subsequent foraging or seeking behaviour [76, 77]. Mathematically, this reflects the fact that cost plays a permissive role, in that it ensures maxima of the ergodic density lie in low-cost regions of sate-space by precluding maxima elsewhere. In this sense, the emphasis is on *autovitiation* of costly fixed points, as opposed to the *autopoiesis* of attractors. 

What we have not addressed here is learning: we have assumed that the agent has already learned the potential energy function *φ*(*x*) that corresponds to environmental constraints on its motion: see [6] for an example of this learning. However, there is a subtle but important point about learning: learning the parameters of the potential function corresponds to learning divergence-free flow, which does not affect the ergodic density or fixed points of the attractor. This contrasts with value-learning, in which divergence-flow is unspecified and the parameters of the value-function are learned. We now look at generalising divergence-based schemes and their role in prescribing sequential and itinerant dynamics.

### 6.2. Optimal Itinerancy and Weakly Attracting Sets

There are clearly many different ways in which we could formulate generalised policies and constrain them with cost-functions: We will concentrate on the use of cost to specify itinerant policies: itinerancy is important because it provides a principled explanation for exploration and foraging in ethology [78]. Furthermore, it provides a key connection to dynamical systems theory approaches to the brain [79] that emphasise the importance of itinerant chaos [80], metastability [81], self-organised critically [82], winnerless competition [83], and attractors [84]. Similar constructs based on metastability have been invoked to understand the dynamics of molecular biology and the emergence of disease states like cancer [85]. The common theme here is the induction of itinerancy though the destruction of fixed-points or the gradients causing them [86]. The ensuing *attractor ruins* or relics [87] provide a framework for heteroclinic orbits that are ubiquitous in neurobiology, in electrophysiology [88], cognition [89], and large-scale neuronal dynamics [90]. 

**Figure 8:**
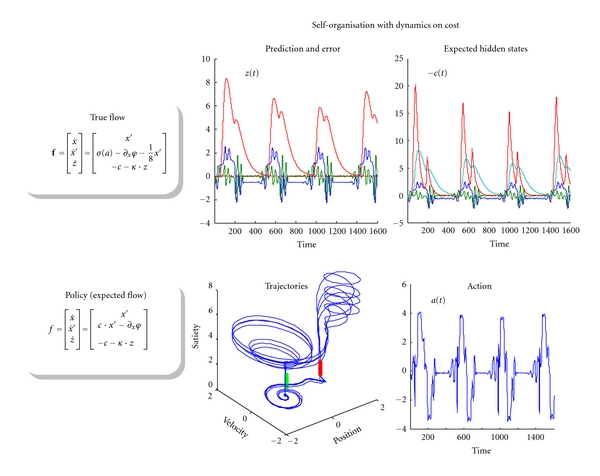
Optimal itinerancy. This figure shows how itinerant dynamics can be constrained by a cost function, leading to a stable heteroclinic channel, in which unstable but attractive fixed points are visited in succession. Here, we have exploited the specification of cost in terms of satiety, which has been made a hidden (physiological) state. This makes cost time dependent and sensitive to the recent history of the agent's states. Placing dynamics on cost enables us to model sequential behaviour elicited by cost functions that are suppressed by the behaviour they elicit. The left panels show the true (upper) and modelled (lower) equations of motion on hidden states, where the latter are constrained by the cost function in Figure 5. Here, satiety increases with rewards (negative cost) and decays with first-order kinetics. The resulting behaviour is summarised in the right-hand side panels. The upper left panel shows the predictions of hidden states and prediction errors; where predictions are based upon the conditional beliefs about hidden states shown on the upper right. These predictions prescribe optimal action (lower right), which leads to the behavioural orbits shown on the lower left. The characteristic feature of the ensuing dynamics is a sequential return to unstable fixed points; denoted by the minimum of the potential landscape (green dots) and the cost-dependent (unstable) fixed point at the target location (red dots).

It is fairly easy to extend the mountain car example above to produce itinerant behaviour with heteroclinic orbits and winnerless competition [83]. Intuitively, this can be regarded as adaptive behaviour in which various rewards are accessed in sequence to maintain physiological homoeostasis (e.g., eating and drinking). This is straightforward to model by making the cost-function state dependent. This enables cost to be changed by the behaviours it induces. A simple example is provided in Figure 8, in which we have made the satiety parameter of the cost function a hidden state in the generative model (and environment) so that satiety increases whenever cost is low. Cost can only be low in attractive states, which means attractive states become unattractive when occupied for too long. In the mountain car setup, when satiety rises, cost is uniformly low everywhere and the agent will simply settle at the bottom of the valley and stay there until satiety decays sufficiently to make the parking location attractive again. Figure 8 shows the equations of motion and ensuing dynamics, using the same format as in previous figures. This behaviour is characteristic of winnerless competition, in the sense that attractive fixed points are inherently unstable and release the trajectory to the next fixed point in the sequence. In this instance, instability is induced dynamically through state-dependent cost. This causes the mountain car to periodically rouse itself from the bottom of the valley and visit the parking location for a short time, until sated and then return to the bottom of the valley for a rest.

The vitiation of costly attractors is a mechanism that appears in several guises and has found important applications in a number of domains. For example, it is closely related to the notion of autopoiesis and self-organisation in situated (embodied) cognition [42]. It is formally related to the destruction of gradients in synergetic treatments of intentionality [86]. Mathematically, it finds a powerful application in universal optimisation schemes [91] and, indeed, as a model of perceptual categorization [92]. The dynamical phenomena, upon which these schemes rest, involve an itinerant wandering through state-space along heteroclinic channels (orbits connecting different fixed points). Crucially, these attracting sets are weak (Milnor) attractors or attractor ruins that expel the state until it finds the next weak attractor. The result is a sequence of transitions through state-space that, in some instances, can be stable and repeating. The resulting stable heteroclinic channels have been proposed as a metaphor for neuronal dynamics and underlying cognitive processing [83]. Furthermore, the notion of Milnor or ruined attractors underlies much of the technical and cognitive literature on itinerant dynamics. For example, one can explain “a range of phenomena in biological vision, such as mental rotation, visual search, and the presence of multiple time scales in adaptation” using the concept of weakly attracting sets [92], see also [93]. It is this sort of dynamical behaviour that may underpin generalised policies that are specified directly in terms of equations of motion (as opposed to value functions in optimal control).

### 6.3. Summary

In this section, we have seen how cost can be used to induce attractive fixed points in hidden state-space while destroying unattractive fixed points. This does not involve any value-learning but rests upon the fact that stable fixed points result from flow with the negative divergence. We have seen how these policies can be realised in a straightforward manner under active inference and how endowing cost with a context sensitivity leads to itinerant but purposeful behaviour that is reminiscent of biological systems. The basic message here is that it may be sufficient to understand adaptive self-organised behaviour purely in terms of the itinerant dynamics induced by an agent's (implicit) prior beliefs about its motion through state-space. These dynamics and their associated attractors can be characterised in terms of unstable fixed points (weak attractors) and, in most instances, an associated sequence of heteroclinic orbits. In this dynamical setting, a natural way to specify (and inherit) the weakly attracting sets that define phenotypic behaviour is to destroy or preclude (stable) fixed points that do not belong to attracting set. Note that this stands in stark contrast to optimal control theory, which tries to optimise the flow using value-functions. However, the (generally intractable) computation of these functions may be unnecessary and unnatural, if it is sufficient to place straightforward constraints on the flow that defines value.

## 7. Discussion

This paper started with the existence of agents (systems) with defining characteristics that are conserved over time. We used arguments from ergodic theory, random dynamical systems and information theory to identify the imperatives for their dynamics. The picture that emerges can be summarised as follows. Agents are equipped (by evolution or engineering) with a characteristic (cost) function of the states they should occupy or possess. This function places constraints on prior beliefs about motion through state-space (state transitions). Action realises this policy by suppressing random or surprising deviations from the ensuing predictions, thereby minimising surprise and the entropy of the ergodic density (over long periods of time). The result is a random dynamical attractor, with small measure that ensures agents occupy a limited attracting set of states (or expresses phenotypic traits that are conserved over time). Every policy (flow) has an associated value-function, which is the (log) ergodic density (or negative surprise) of any generalised state. The self-consistency of cost-functions and the ergodic densities they engender is assured by natural selection; in the sense that cost-functions that do not induce ergodic behaviour cannot be inherited. In the final sections, we compared and contrasted policies from optimal control theory with generalised policies based on dynamical systems theory that lead to itinerant behaviour.

### 7.1. Dynamics versus Reinforcement-Learning

The formulations in this paper emphasise the link between cost-functions and policies. Optimal control theory and reinforcement-learning assumes value is expected cost in the future. This enables the policy to be optimised in terms of control, such that the expected path integral of cost is minimised. The ensuing policies are prescribed directly by value, which acts as a guiding function. This entails finding a solution to a set of self-consistent equations linking value and cost. However, this is a difficult problem and leads to some inconsistencies; for example, the autonomous or random explorations of state-space needed to furnish solutions to the Bellman equations are precisely the fluctuations that optimal control is trying to avoid. Generalised policies resolve these difficulties because they do not define value as expected cost: value is defined in terms of the states that are visited most frequently (i.e., the ergodic density), and is a function of flow (the policy). The last section tried to show that there are straightforward ways to place constrain policies; namely, to destroy unattractive fixed points. In summary, reinforcement-learning starts with a cost-function from which the value-function is derived. The value is then used to optimise a policy. Conversely, in the setting of random attractors, cost-functions constrain the policy directly. By definition, the policy then maximises value or minimises surprise. This eschews the solution of the appropriate Bellman equation, provides a principled explanation for exploratory or itinerant dynamics, and affords an efficient and biologically plausible scheme. Furthermore, it allows action to actively suppress unpredicted deviations from the policy.

The importance of dynamical itinerancy has been articulated many times in the past [94], particularly from the perspective of computation and autonomy; see [93] for a focus on Milnor attractors. It has also been considered formally in relation to cognition; see [87] for a focus on attractor relics, ghosts, or ruins. Indeed, there is growing interest in understanding brain dynamics *per se* in terms of itinerancy and metastability [81, 83, 88, 89]. Tani et al., [95] consider itinerant dynamics in terms of bifurcation parameters that generate multiple goal-directed actions (on the behavioural side) and optimization of the same parameters (when recognizing actions). They provide a series of elegant robotic simulations to show generalization by learning with this scheme. See also [96] for interesting simulations of itinerant exploration, using just prediction errors on sensory samples over time.

Reinforcement-learning frames the problem of adaptive behaviour in terms of accessing distal and sparse rewards. In one sense this is not a problem; it is the solution entailed by an agent and its interactions with the environment. In this view, agents do not seek out valuable (rewarding) states; valuable states are just states the agent frequents. This challenges any preconception that optimal control has a central or unique role in adaptive behaviour. Having said this, the premise of optimal control and reinforcement-learning that agents minimise expected future costs is a compelling and enduring heuristic. This heuristic may be exploited by the brain, particularly in terms of high-level (e.g., cognitive) processing using model-based schemes.

### 7.2. Value-Learning versus Perceptual Learning

The mountain car example can be regarded as a model of behavioural responses constrained by *innate or formal* priors (cost-functions). However, most of the interesting issues in a biological setting may rest on *acquired or empirical* priors that are optimised during perception. Irrespective of the details of this optimisation, under active inference, optimisation becomes a perceptual inference and learning problem. In other words, the notion that stimulus-response links are selectively reinforced during learning disappears and is replaced by the learning of stimulus-stimulus associations. These prescribe conditional beliefs, which action fulfills. This is important because it places much of behaviourism in the domain of perception and reiterates the close links between action and perception. 

It is well know from the complete class theorem that there is a close relationship between priors and cost-functions; in the sense that any admissible decision rule is Bayes-optimal for at least one prior and cost-function [97]. The treatment in this paper suggests that when decisions involve inferred states of the world, cost-functions can be treated as priors. Heuristically, cost-functions are a fixed attribute of the agent and can therefore only manifest as formal priors on the agent's inference and consequent behaviour. This is particularly important in a biological setting, where natural selection endows agents with formal or innate priors that constrain their exchanges with the environment.

## 8. Conclusion

In this paper, we have tried to understand optimal control theory in relation to the free energy principle. We started with a review of ensemble dynamics and the perspective it provides on reinforcement-learning and optimal control. These approaches furnish policies or equations of motion that converge on attractors in state-space that are specified by a cost-function. Conventional schemes specify these equations in terms of value-functions or cost-to-go, which entail the solution of the appropriate Bellman equation. We considered a dynamical alternative based on the selective destruction of stable fixed-points in costly regimes of state-space. Although less efficient at minimising the path integral of cost, this divergence-based scheme involves no value-learning and accounts for exploratory dynamics that do not need stochastic interventions. In this context, the policies of optimal control become formal priors in generative models used to infer hidden states and predict sensations. Action fulfils these predictions by suppressing a free energy bound on surprise. Crucially, optimising action, perceptual inference, perceptual learning, and the priors themselves are all mandated by the free energy principle. This principle is simply a restatement of the fact that adaptive systems resist a natural tendency to disorder. In summary, agents must be equipped with policies that prescribe their expected motion through state-space (i.e., state transitions) and the ability to counter surprising (random) deviations from the expected trajectory though action. The motion prescribed by the policy (and realised by action) induces low entropy densities (in terms of ensemble dynamics) or random attractors with small measure (in terms of random dynamical systems). These are sufficient to explain to existent of ergodic self-organising systems, whose attributes are conserved over time. 

## Appendices

### A. Entropy Production

This appendix shows that the dispersion of states *x* ∈ *X* by random fluctuation increases entropy. This implies (autopoietic) flow must reduce entropy to maintain an invariance probability density over states. This is based on the following lemma.

Lemma A.1 (entropy production) The entropy production of a differentiable dynamical system (*T*, *X*, *f*) can be decomposed into a flow and non-negative dispersion term

(A.1) $$\dot{ℋ}\left(X\right)=\int_{X}\nabla \cdot \left(fp\right)\ln p\left(x\right)dx+\int_{X}\frac{\nabla p\cdot \Gamma \cdot \nabla p}{p\left(x\right)}dx.$$

Proof We start with the Fokker-Planck equation describing the rate of change of an ensemble density *p*(*x*, *t*) in terms of dispersion due to random fluctuations and flow *f* : *X* → *X*,

(A.2) $$\dot{p}\left(x,t\right)=\nabla \cdot \Gamma \nabla p-\nabla \cdot \left(fp\right).$$

Without loss of generality, we assume some diffeomorphism of state-space in which the diffusion tensor can be expressed as Γ(*x*) = *γ*(*x*)*I*, where *γ*(*x*) ∈ ℝ^+^. The existence of this state-space is assured by the positive definiteness of the diffusion tensor. The entropy is

(A.3) $$\begin{array}{c} ℋ\left(X\right)=-\int p\ln pdx\Rightarrow \\ \dot{ℋ}\left(X\right)=-\int \dot{p}\ln pdx-\int \dot{p}dx=-\int \dot{p}\ln pdx. \end{array}$$

The second term in the expression for entropy production $\dot{ℋ}(X)$ disappears because probability mass is conserved. The rate of change of entropy can be decomposed into flow- and dispersion-dependent terms by substituting (A.2) into (A.3)

(A.4) $$\dot{ℋ}\left(X\right)=\int \nabla \cdot \left(fp\right)\ln pdx-\gamma \int \left(\nabla^{2}p\right)\ln pdx.$$

Finally, the self-adjoint property of the Laplacian operator ∇^2^ means that the second (dispersion) term is greater or equal to zero:

(A.5) $$\begin{array}{cc} \int \left(\nabla^{2}p\right)\ln pdx & =\int p(\nabla^{2}\ln p)dx\\ & =\int \left(\nabla^{2}p\right)dx-\int \frac{\nabla p\cdot \nabla p}{p}dx\\ & =-\int \frac{\nabla p\cdot \nabla p}{p}dx\leq 0. \end{array}$$

The integral of the curvature ∇^2^ *p* disappears because *p* : = *p*(*x*, *t*) is a proper density. Substituting (A.5) into (A.4) gives (A.1).

Remark A.2 s Equation (A.5) means that random fluctuations increase entropy in proportion to their amplitude and the roughness of the ensemble density. In the absence of deterministic flow, the entropy increases until the density has dispersed and its gradients disappear; that is, $\nabla p=0\Rightarrow \dot{ℋ}(X)=0$. In the presence of flow, (A.4) implies, at steady state $\dot{ℋ}(X)=0$ and flow decreases entropy to offset dispersion-related increases. See Tomé [41] for a fuller treatment in terms of probability currents.

### B. Sensory Entropy

This appendix shows that the entropy of hidden states is bounded by the entropy of sensory states. This means that if the entropy of generalised sensory signals is minimised, so is the entropy of the hidden states that caused them. We will assume sensory states are an analytic function of hidden states plus some fluctuations. This implies (in generalised coordinates of motion) that

(B.1) $$\begin{array}{c} \begin{array}{c} s=g\left(x\right)+\omega_{s}\\ \dot{x}=f\left(x\right)+\omega_{x} \end{array}\Rightarrow \left[\begin{array}{c} s\\ s^{\prime }\\ s^{\prime \prime }\\ \vdots \end{array}\right]=\left[\begin{array}{c} g\left(x\right)\\ \partial_{x}g\cdot x^{\prime }\\ \partial_{x}g\cdot x^{\prime \prime }\\ \vdots \end{array}\right]+\left[\begin{array}{c} \omega_{s}\\ \omega_{s}^{\prime }\\ \omega_{s}^{\prime \prime }\\ \vdots \end{array}\right] \end{array},$$

where the second equability can be written more compactly as $\tilde{s}=\tilde{g}(\tilde{x})+\tilde{\omega }$.

Lemma B.1 (entropy bounds) If the sensitivity of the sensory mapping $|\partial \tilde{g}/\partial \tilde{x}|$ is uniform over hidden states, then the entropy of hidden states is bounded by the entropy of sensory states, to within an additive constant:

(B.2) $$H\left(S\;|\;m\right)\geq H\left(X\;|\;m\right)+\ln\left|\frac{\partial \tilde{g}}{\partial \tilde{x}}\right|.$$

Proof Because the random fluctuations are conditionally independent, we have (Theorem 6.5 in [98, page 151]) the following:

(B.3) $$H\left(\tilde{S}\;|\;m\right)\geq H\left(\tilde{X}\;|\;m\right)+\int p\left(\tilde{x}\;|\;m\right)\ln\left|\frac{\partial \tilde{g}}{\partial \tilde{x}}\right|d\tilde{x}.$$

Because the log sensitivity is constant, it can be placed outside the integral, which integrates to unit, giving (B.2).

Remark B.2 s Here. $\partial \tilde{g}/\partial \tilde{x}$ is the sensitivity or gradient of the generalised sensory mapping with respect to the hidden states. The integral in (B.2) reflects the fact that entropy is not invariant to a change of variables and assumes that the sensory mapping $\tilde{g}:X\to S$ is diffeomorphic (i.e., bijective and smooth). This requires the hidden and sensory state-spaces to have the same dimension, which can be assured by truncating generalised states at an appropriate order to make dim⁡(*S* | *m*) = dim⁡(*X* | *m*).

### C. The Laplace Assumption

This appendix shows why it is only necessary to retain the conditional mean under a fixed-form Laplace assumption about the recognition density. If we assume $q(\vartheta )=𝒩(\tilde{\mu },\Sigma )$ is Gaussian (the Laplace assumption), then we can express free energy in terms of its sufficient statistics; the mean and covariance, $\left(\tilde{\mu },\Sigma \right)$ using Gibb's energy $U(t)=-\ln p(\tilde{s},\vartheta |m)$ and $n=\dim(\tilde{\mu })$

(C.1) $$ℱ=U\left(\tilde{\mu }\right)+\frac{1}{2}\mathrm{tr}\left(\Sigma \frac{\partial }{\partial {\tilde{\mu }}^{2}}U\right)-\frac{1}{2}\ln\left|\Sigma \right|-\frac{n}{2}\ln2\pi e.$$

We can now minimise free-energy with respect to the conditional precision Π = Σ^−1^ by solving ∂_Σ_ *F* = 0 to give

(C.2) $$\partial_{\Sigma }ℱ=-\frac{1}{2}\Pi +\frac{1}{2}\frac{\partial }{\partial {\tilde{\mu }}^{2}}U=0\Rightarrow \Pi =\frac{\partial^{2}}{\partial {\tilde{\mu }}^{2}}U\left(\tilde{\mu }\right).$$

Critically, this is an analytic function of the mean and does not have to be evaluated explicitly. Furthermore, we can simplify the expression for free-energy by eliminating the curvature from (C.1) to give *ℱ* = *U*(*μ*) − (1/2)ln⁡|Σ| − (*n*/2)ln⁡2*π*.

### D. Value and Ergodic Densities

For simplicity, we will ignore the state-dependence of *Q*(*x*) and assume it is constant; a full treatment can be found in [58].

Lemma D.1 (ergodic density) The ergodic density of a dynamical system with Fokker Planck operator Λ = ∇·(Γ∇−*f*) and flow *f* = (Γ + *Q*)∇*V* subject to *Q* = −*Q* ^*T*^ is given by

(D.1) p(x ∣ m)=exp⁡(V)⇒∇p=p∇V⇒Λp=0.

Proof By substituting ∇*p* = *p*∇*V* and *f* = Γ∇*V* + *Q*∇*V* into the Fokker Planck operator we get

(D.2) $$\begin{array}{cc} \Lambda p & =\nabla \cdot \Gamma \nabla p-\nabla \cdot \left(fp\right)\\ & =\nabla \cdot \left(p\Gamma \nabla V\right)-\nabla \cdot \left(p\Gamma \nabla V\right)-\nabla \cdot \left(pQ\nabla V\right)\\ & =-p\nabla \cdot \left(Q\nabla V\right)-\left(Q\nabla V\right)\cdot \nabla p\\ & =-p\left(\nabla \cdot \left(Q\nabla V\right)+\left(Q\nabla V\right)\cdot \nabla V\right) \end{array}$$

one can see that (D.2) is satisfied when the second component of flow *Q*∇*V* is divergence free and orthogonal to ∇*V*. Given *Q* = −*Q* ^*T*^, it is easy to see both these conditions are met

(D.3) $$\begin{array}{c} \nabla \cdot \left(Q\nabla V\right)=\mathrm{tr}\left(Q\partial_{xx}V\right)=0,\\ \nabla V\cdot \left(Q\nabla V\right)=\mathrm{tr}\left(Q\partial_{x}V\partial_{x}V^{T}\right)=0. \end{array}$$

This means that *p* = exp⁡(*V*) is the ergodic density or eigensolution Λ*p* = 0 of the Fokker-Planck operator describing density dynamics.

### E. Cost Functions and Value

Here, we derive cost as a function of value, using discrete and continuous time formulations.

Discrete Case For the discrete case, let **c** (resp., **v**) be a row-vector of the cost (resp., value) of every discrete state *x*_*t*_ ∈ *X* at time *t* and **P**_*ij*_ = *p*(*x*_*t*+1_ = *i* | *x*_*t*_ = *j*) be the transition probability or policy. By definition, value is (assuming **c** **P** ^*∞*^ = 0)

(E.1) $$\begin{array}{cc} v & =-\overset{\infty }{\underset{t=0}{\sum }}cP^{t}\Rightarrow \\ vP & =-\overset{\infty }{\underset{t=1}{\sum }}cP^{t}=v+c\Rightarrow c=vP-v=v_{1}-v_{0}. \end{array}$$

The subscripted **v**_*t*_ : = **v** **P** ^*t*^ is the value expected at time step *t* in the future. It can be seen that cost is just the expected increase in value over the next time step.

Continuous Case In the continuous time case, the value of *x*_0_ ∈ *X* is the cost expected over future times *t* ∈ (*t*_0_, *∞*], starting with a point density, *p*(*x*, *t*_0_ | *m*) = *δ*(*x*_0_). In the infinite horizon case, assuming the cost expected at equilibrium is zero; that is,

(E.2) $$\begin{array}{cc} & \overset{}{\underset{x\in X}{\int }}c\left(x\right)p\left(x\;|\;m\right)dx=0 \end{array}.$$

We have, by definition

(E.3) $$\begin{array}{cc} V\left(x_{0}\right) & =-\overset{\infty }{\underset{t_{0}}{\int }}\overset{}{\underset{x\in X}{\int }}c\left(x\right)p\left(x,t\;|\;m\right)dx\;dt\Rightarrow \\ \dot{V}\left(x_{0}\right) & =\int c\left(x\right)p\left(x,t_{0}\;|\;m\right)dx=c\left(x_{0}\right)\\ & =\int V\left(x\right)\dot{p}\left(x,t_{0}\;|\;m\right)dx\\ & =\int V\left(x\right)(\nabla \cdot \Gamma \cdot \nabla \delta \left(x_{0}\right)-f\cdot \nabla \delta \left(x_{0}\right)\\ & \;\;\;\;\;-\delta \left(x_{0}\right)\nabla \cdot f)dx\\ & =\nabla \cdot \Gamma \cdot \nabla V\left(x_{0}\right)+\nabla \cdot \left(V\left(x_{0}\right)f\right)\\ & \;-V\left(x_{0}\right)\nabla \cdot f\Rightarrow \\ c\left(x\right) & =f\cdot \nabla V\left(x\right)+\nabla \cdot \Gamma \cdot \nabla V\left(x\right)=\dot{V}\left(x\right). \end{array}$$

This says that the increase in value expected over time is the cost at any location in state-space. However, this is also the increase in expected value over state-space, which can be expressed using the Fokker-Planck equation (A.2). Evaluating this expression at the initial density shows that cost comprises two components; one due to deterministic flow and another to dispersion (the first and second terms in the last equality).

### F. Optimal Control and Policies

This appendix provides a brief review of classical formulations of optimal control in discrete and continuous time. For simplicity, we will ignore random fluctuations in the continuous case. The value-function prescribes an optimal policy, which can be expressed in discrete and continuous form as

(F.1) $$\begin{array}{c} \pi^{*}=\underset{\pi }{\arg\;\max\;}vP\left(\pi \right),\\ \pi^{*}=\underset{\pi }{\arg\;\max}\;f\left(x,\pi \right)\cdot \nabla V\left(x\right), \end{array}$$

where *π* parameterises the transition probability matrix in the discrete case and *u* = *π*(*x*) represents a mapping between states and control in the continuous case. Equation (F.1) means that the optimal policy **P**(*π**) maximises the expected value of the next state. This is the solution to the Bellman and Hamilton-Jacobi-Bellman equations for the discrete and continuous cases, respectively,

(F.2) $$\begin{array}{c} c=\underset{\pi }{\max}\left\{vP\left(\pi \right)-v\right\},\\ c\left(x\right)=\underset{\pi }{\max}\left\{f\left(x,\pi \right)\cdot \nabla V\left(x\right)\right\}. \end{array}$$

More generic forms of the Bellman and HJB equations consider loss incurred by the state-transitions *per se*, in which case the cost-function is brought into the maximum operator

(F.3) $$\begin{array}{c} \underset{\pi }{\max}\left\{vP\left(\pi \right)-c\left(\pi \right)-v\right\}=0,\\ \underset{\pi }{\max}\left\{f\left(x,\pi \right)\cdot \nabla V-c\left(x,\pi \right)\right\}=0. \end{array}$$

Furthermore, one might want to consider cost-functions that change with time giving, for the continuous case

(F.4) $$\underset{\pi }{\max}\left\{f\left(x,\pi \right)\cdot \nabla V-c\left(x,\pi \right)\right\}=-\dot{V}.$$

Although important cases in engineering problems, these generalisations are not so interesting from the point of view of ensemble dynamics with ergodicity (especially when one considers cost-functions on generalised states). However, in differential game theory, the HJB equation generalizes to the Hamilton-Jacobi-Isaacs (HJI) equations [99].

### G. Value Learning and Optimality Equations

This appendix reviews standard solutions to the Bellman (discrete) and HJB (continuous) equations based on the Robbins-Monro algorithm (or stochastic iteration algorithm [67]) for solving systems of the form **v** = *h*(**v**),

(G.1) v⟵v+ρδ:δ=h(v)−v.

Here, *δ* corresponds to a prediction error and *ρ* ∈ [0,1] determines the convergence rate.

Discrete Case Using *h*(**v**) = **v** **P**(*π**) − **c** from (F.3), one could optimize the value-function of all states simultaneously using (G.1) [100]. This is an example of a *model-based* scheme because it rests on a model of state-transitions implicit in the policy **P**(*π*). Simpler *model-free* schemes adopt a stochastic or sampling approach to approximating the value-function with an estimate *v*(*x*_*t*_) of each state visited

(G.2) $$v\left(x_{t}\right)⟵v\left(x_{t}\right)+\rho \delta_{t}:\delta_{t}=v\left(x_{t+1}\right)-v\left(x_{t}\right)-c\left(x_{t}\right).$$

Here, *δ* corresponds to a reward prediction error. When its average converges to zero, then the long-term average **v**_*i*_ = 〈*v*(*x*_*i*_)〉_*t*_ is the desired solution to the Bellman equation,

(G.3) $${\langle \delta_{t}\rangle }_{t}=0\Rightarrow c\left(x_{t}\right)={\langle v\left(x_{t+1}\right)-v\left(x_{t}\right)\rangle }_{t}\Rightarrow c=vP\left(\pi^{*}\right)-v.$$

The variance of *v*(*x*_*i*_) can be made arbitrary small by decreasing *ρ*. Q-learning rests on a similar scheme, but replaces value with quality *Q* : *X* × *U* → ℝ on the joint space of states and action

(G.4) $$\begin{array}{cc} Q\left(x_{t},u_{t}\right)⟵Q\left(x_{t},u_{t}\right)+\rho \delta :\delta & =\underset{u}{\max}Q\left(x_{t+1},u\right)-Q\left(x_{t},u_{t}\right)\\ & \;-c\left(x_{t},u_{t}\right). \end{array}$$

From this stochastic value iteration scheme, one can calculate expected reward for any state-action pair. A related state-action-reward-state-action scheme [101] is

(G.5) $$\begin{array}{cc} Q\left(x_{t},u_{t}\right)⟵Q\left(x_{t},u_{t}\right)+\rho \delta :\delta & =Q\left(x_{t+1},u_{t+1}\right)-Q\left(x_{t},u_{t}\right)\\ & \;-c\left(x_{t},u_{t}\right). \end{array}$$

This learns the Q-values associated with taking the policy it follows while Q-learning learns the Q-values associated with the optimal policy. While these are important generalisations, one can simplify things theoretically by replacing Q-values with a value-function on the Cartesian product of state and action spaces; that is, *Q*(*x*, *u*) ≡ *V*(*x*) : *x* ∈ *X* × *U*. This implicit factorisation of state-space has been used for approximating value-functions in Markov decision processes with large state and action spaces [102].

Continuous Case A neurobiologically plausible continuous-time scheme was introduced in [72]. This scheme exploits the fact that the derivative of the value-functional (time-varying value of the current state) is just cost $\dot{V}(x(t))=c(x)$ (in the deterministic limit of (E.3)). Here, the prediction-error or teaching signal $\delta ={\dot{\upsilon }}_{c}+{\dot{\upsilon }}_{a}$ is the derivative of *innate* and *acquired* value (e.g., the phasic responses of dopaminergic or cholinergic neurons). Innate value is the antiderivative of reward ${\dot{\upsilon }}_{c}=-c(x)$ registered by specific neuronal systems (e.g., the lateral hypothalamic area). Acquired value *υ*_*a*_ is parameterised by connection strengths coupling sensory states to the neuronal structures that encode it (e.g., the amygdala). Connection strengths mediating acquired value and stimulus-response links are optimized by conventional associative plasticity. Crucially, this plasticity is modulated or enabled by the teaching signal. After convergence,

(G.6) $$\delta ={\dot{\upsilon }}_{c}+{\dot{\upsilon }}_{a}=0\Rightarrow {\dot{\upsilon }}_{a}=-{\dot{\upsilon }}_{c}=c\left(x\right)=\dot{V}\left(x\left(t\right)\right),$$

the acquired value becomes the true value ${\dot{\upsilon }}_{a}=\dot{V}(x(t))$ (to within an additive constant).

### H. Integrating Active Inference Schemes

The simulations in this paper involve integrating time-varying states in both the environment and the agent as a single system, which can be modelled with the following ordinary differential equation, where random fluctuations enter as analytic forcing terms and defining $\tilde{f}:=f(\tilde{x},a,\theta )$:

(H.1) $$\dot{u}=\left[\begin{array}{c} \dot{\tilde{s}}\\ \dot{\tilde{x}}\\ {\dot{\tilde{\omega }}}_{s}\\ {\dot{\tilde{\omega }}}_{x}\\ \dot{\tilde{\mu }}\\ \dot{a} \end{array}\right]=\left[\begin{array}{c} 𝒟\tilde{g}+𝒟{\tilde{\omega }}_{s}\\ \tilde{f}+{\tilde{\omega }}_{x}\\ 𝒟{\tilde{\omega }}_{s}\\ 𝒟{\tilde{\omega }}_{x}\\ 𝒟\tilde{\mu }-\partial_{\tilde{\mu }}ℱ\\ -\partial_{a}ℱ \end{array}\right].$$

To update these collective states we use a local linearization; $\Delta u=(\exp(\Delta tℑ)-I)ℑ{\left(t\right)}^{-1}\dot{u}$ over time steps of Δ*t*, where

(H.2) $$\begin{array}{cc} ℑ=\frac{\partial \dot{u}}{\partial u}= & \left[\begin{array}{cccccc} 0 & 𝒟\partial_{\tilde{x}}\tilde{g} & 𝒟 & & & 𝒟\partial_{a}\tilde{g}\\ & \partial_{\tilde{x}}\tilde{f} & & I & & \partial_{a}\tilde{f}\\ & & 𝒟 & & & \\ & & & 𝒟 & & \\ -\partial_{\tilde{\mu }\tilde{s}}ℱ & & & & 𝒟-\partial_{\tilde{\mu }\tilde{\mu }}ℱ & -\partial_{\tilde{\mu }a}ℱ\\ -\partial_{a\tilde{s}}ℱ & & & & -\partial_{a\tilde{\mu }}ℱ & -\partial_{aa}ℱ \end{array}\right] \end{array}.$$

Because action can only affect free energy through sensory data, it can only affect sensory prediction error. Therefore action dynamics are as follows:

(H.3) $$\begin{array}{c} \dot{a}=-\partial_{a}ℱ=-\partial_{a}\tilde{s}\cdot {\tilde{\varepsilon }}_{s},\\ \partial_{a}\tilde{s}=\partial_{\tilde{x}}\tilde{g}\sum_{i}{𝒟}^{-i}{\left(\partial_{\tilde{x}}\tilde{f}\right)}^{i-1}\partial_{a}\tilde{f}. \end{array}$$

The partial derivative of the generalised sensory data with respect to action depends on changes in the generalised motion of hidden states and is specified by the generative process. Note that this partial derivative depends on the unknown generative process. However, sensations are generally produced in a simple and direct way by action (e.g., proprioception with stretch receptors), such that the dependency can be treated as known.

These equations may look complicated but can be evaluated automatically using numerical derivatives. All the simulations in this paper used just one routine—**spm_ADEM.m**. Demonstrations of this scheme are available as part of the SPM software (http://www.fil.ion.ucl.ac.uk/spm; **DEM_demo.m**), which reproduces the example shown in Figure 7.
